# Targeting a vulnerable septum-hippocampus cholinergic circuit in a critical time window ameliorates tau-impaired memory consolidation

**DOI:** 10.1186/s13024-023-00614-7

**Published:** 2023-04-14

**Authors:** Dongqin Wu, Nana Yu, Yang Gao, Rui Xiong, Luping Liu, Huiyang Lei, Sen Jin, Jiale Liu, Yingzhou Liu, Jiazhao Xie, Enjie Liu, Qiuzhi Zhou, Yanchao Liu, Shihong Li, Linyu Wei, Jingru Lv, Huilin Yu, Wenbo Zeng, Qiang Zhou, Fuqiang Xu, Min-Hua Luo, Yao Zhang, Ying Yang, Jian-Zhi Wang

**Affiliations:** 1 Department of Pathophysiology, School of Basic Medicine, Key Laboratory of Education Ministry of China/Hubei Province for Neurological Disorders, Tongji Medical College, Huazhong University of Science and Technology, Wuhan, 430030 China; 2 Department of Pathology, Union Hospital, Tongji Medical College, Huazhong University of Science and Technology, Wuhan, 430022 China; 3 School of Biomedical Sciences, Faculty of Medicine, The Chinese University of Hong Kong, Shatin, NT, Hong Kong SAR, 999077 China; 4 The Brain Cognition and Brain Disease Institute (BCBDI), Shenzhen Institute of Advanced Technology, Chinese Academy of Sciences, Shenzhen-Hong Kong Institute of Brain Science-Shenzhen Fundamental Research Institutions, Shenzhen, 518055 China; 5 State Key Laboratory of Virology, CAS Center for Excellence in Brain Science and Intelligence Technology (CEBSIT), Wuhan Institute of Virology, Chinese Academy of Sciences, Wuhan, 430071 China; 6 State Key Laboratory of Chemical Oncogenomics, Guangdong Provincial Key Laboratory of Chemical Genomics, Peking University Shenzhen Graduate School, Shenzhen, 518055 China; 7 Center for Excellence in Brain Science and Intelligence Technology, Chinese Academy of Sciences, Shanghai, 200031 China; 8 Endocrine Department of Liyuan Hospital, Tongji Medical College, Huazhong University of Science and Technology, Wuhan, 430077 China; 9 Co-innovation Center of Neuroregeneration, Nantong University, Nantong, 226000 China

**Keywords:** Medial septum, Hippocampus, Cholinergic circuit, Tau, Spatial memory, Alzheimer disease

## Abstract

**Background:**

Abnormal tau accumulation and cholinergic degeneration are hallmark pathologies in the brains of patients with Alzheimer’s disease (AD). However, the sensitivity of cholinergic neurons to AD-like tau accumulation and strategies to ameliorate tau-disrupted spatial memory in terms of neural circuits still remain elusive.

**Methods:**

To investigate the effect and mechanism of the cholinergic circuit in Alzheimer's disease-related hippocampal memory, overexpression of human wild-type Tau (hTau) in medial septum (MS)-hippocampus (HP) cholinergic was achieved by specifically injecting pAAV-EF1α-DIO-hTau-eGFP virus into the MS of ChAT-Cre mice. Immunostaining, behavioral analysis and optogenetic activation experiments were used to detect the effect of hTau accumulation on cholinergic neurons and the MS-CA1 cholinergic circuit. Patch-clamp recordings and in vivo local field potential recordings were used to analyze the influence of hTau on the electrical signals of cholinergic neurons and the activity of cholinergic neural circuit networks. Optogenetic activation combined with cholinergic receptor blocker was used to detect the role of cholinergic receptors in spatial memory.

**Results:**

In the present study, we found that cholinergic neurons with an asymmetric discharge characteristic in the MS-hippocampal CA1 pathway are vulnerable to tau accumulation. In addition to an inhibitory effect on neuronal excitability, theta synchronization between the MS and CA1 subsets was significantly disrupted during memory consolidation after overexpressing hTau in the MS. Photoactivating MS-CA1 cholinergic inputs within a critical 3 h time window during memory consolidation efficiently improved tau-induced spatial memory deficits in a theta rhythm-dependent manner.

**Conclusions:**

Our study not only reveals the vulnerability of a novel MS-CA1 cholinergic circuit to AD-like tau accumulation but also provides a rhythm- and time window-dependent strategy to target the MS-CA1 cholinergic circuit, thereby rescuing tau-induced spatial cognitive functions.

**Graphical Abstract:**

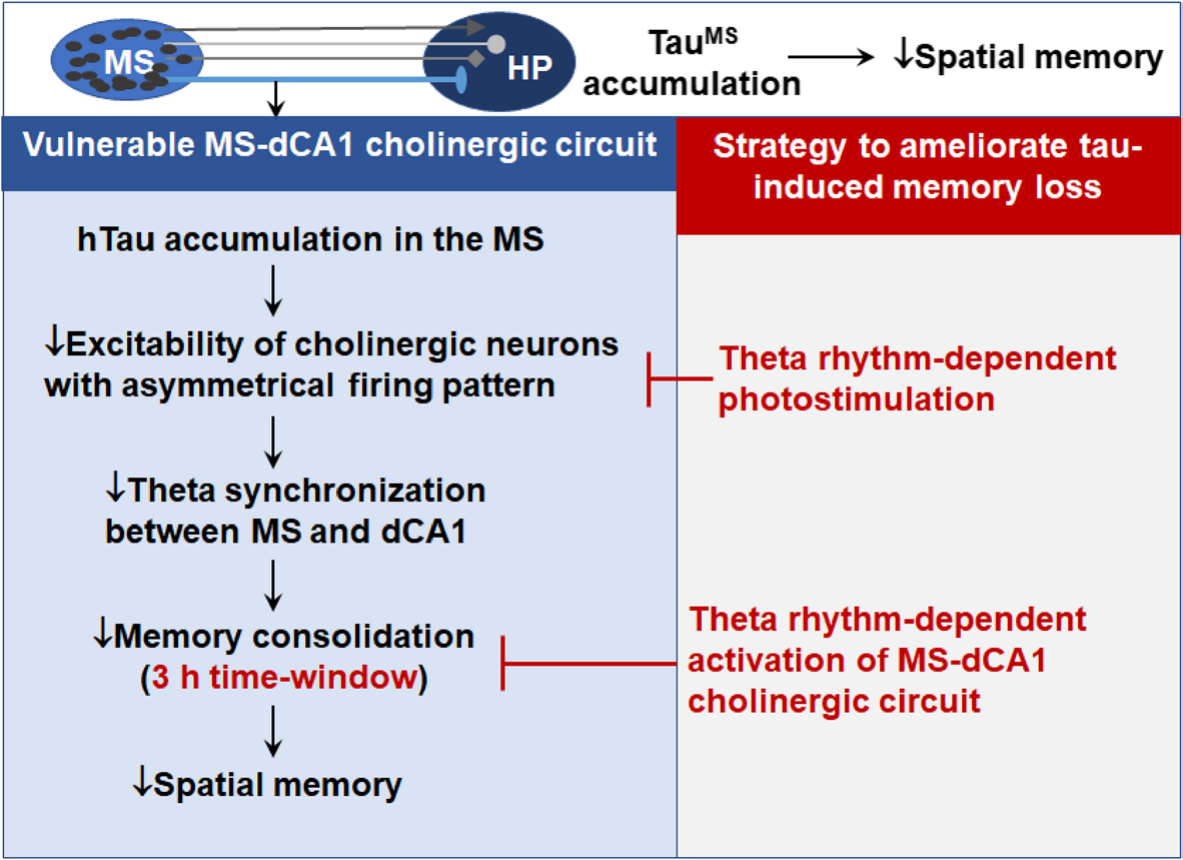

**Supplementary Information:**

The online version contains supplementary material available at 10.1186/s13024-023-00614-7.

## Background

The accumulation of abnormal Tau in the human brains has been implicated in major neurodegenerative diseases termed tauopathies, including Alzheimer’s disease (AD), Parkinson’s disease (PD), frontotemporal dementia with Parkinsonism-17 (FTDP-17), and so on [1–3]. A positive correlation between increased tau pathology and the severity of memory deficits in AD indicates that tau accumulation is a direct mediator of neurodegenerative disease [4–7].

Cholinergic innervations undergo severe neurodegeneration during AD progression [8, 9]. Loss of basal forebrain cholinergic neurons projecting to cortical structures contributes to cognitive impairment in patients with AD [10]. In the clinic, treatments aimed at improving cholinergic function, such as cholinesterase inhibitor therapies, remain the most commonly used for AD patients [11, 12]. Basal forebrain cholinergic systems constitute discrete cell groups that innervate numerous brain regions. For instance, neurons in the medial septum (MS) and vertical diagonal band of Broca (vDB) predominantly extend their axons to the hippocampus, forming the septo-hippocampal pathways [13, 14]. However, whether and which MS-hippocampus cholinergic pathway is vulnerable to tau accumulation remain enigmatic. Moreover, there is an urgent need to study the targeting strategy for the MS-hippocampus cholinergic pathway to rescue tau-impaired memory.

In the present study, we dissected the MS-CA1 cholinergic connection and outlined its time-dependent degeneration induced by hTau accumulation in the MS. We found that tau accumulation within MS cholinergic neurons produced robust inhibition of cholinergic neurons with asymmetric firing characteristics, especially in the MS-CA1 cholinergic pathway. During the first 3 h time window of memory consolidation, photoactivation of MS cholinergic neurons or MS-CA1 cholinergic inputs could efficiently rescue tau-induced spatial memory decline in a strict theta-rhythm-dependent manner. These beneficial effects were completely abolished by the administration of blocker cocktails containing nicotinic and muscarinic acetylcholine receptor antagonists in the CA1. Our findings reveal a novel circuit mechanism by which MS tau accumulation disrupts memory consolidation, resulting in spatial memory loss. Intriguingly, stimulation of the MS-CA1 cholinergic neural circuit at theta rhythm exerts promising therapeutic efficiency on tau-impaired spatial memory, especially within a critical 3 h time window after spatial learning.

## Materials and methods

### Animals

The 3xTg mouse (Stock No:34830, 129S4.CgTg (APPSwe, tauP301L) 1Lfa*Psen1 *^*tm1Mpm*^ /Mmjax) was a gift from Prof. Xifei Yang (Shenzhen Center for Disease Control and Prevention). The 5xFAD mouse (Stock No:034840, B6SJL-Tg (APPSwFlLon, PSEN1*M146L*L286V) 6799Vas/Mmjax) was from Prof. Bai Lu (School of Medicine, Tsinghua University, Beijing, China). The ChAT-Cre mouse (Stock No:006410, ChAT-IRES-Cre: SV40pA:frt-neo-frt (Chattm2 (cre) Lowl) was from Prof. You Wan (Peking University, Beijing, China) and bred in the SPF-level laboratory animal room. C57BL/6 mice (male, 12 weeks) were purchased from Vital River Animal Technology Company in Beijing. The genotypes of the mice were identified by polymerase chain reaction (PCR) analysis of toe DNA. All mice were bred and grown in SPF condition with free access to food and water and were housed in four to five littermates with a stable circadian rhythm (7:00–19:00 lights on; 19:00–7:00 lights off). All experiments were conducted in accordance with the Tongji Medical College Guide for the Care and Use of Laboratory Animals and with the approval of the Institutional Animal Care and Use Committee of Huazhong University of Science & Technology.

### Virus vectors

The pAAV-CAG-Vector/hTau-eGFP, pAAV-EF1α-DIO-Vector-eGFP, pAAV-EF1α-DIO-hTau-eGFP and pAAV-EF1α-DIO-hTau-mCherry were constructed and packaged based on the plasmids EGFP-tau-2N4R and EGFP-vector-2N4R by Obio Technology (Shanghai, China). pAAV-EF1α-DIO-ChR2(H134R)-eYFP / mCherry, pAAV-EF1α-DIO-eYFP, pAAV-CaMKII-Cre-mCherry, and pAAV-EF1α-DIO-mCherry were purchased from Obio Technology. AAV viruses were serotyped with AAV8 coat proteins. H129-G4 [15, 16] was a gift from Prof. Min-Hua Luo.

### Antibodies and reagents

Anti-pT205 (SAB,11108-2), anti-pT231 (SAB,11110), anti-HT7 (Thermo, MN1000), anti-Tau5(Abcam, ab80579), β-actin (Abcam, ab6276), ChAT (Chemicon, AB144P), CaMKII (GeneTex, GTX127939), GABA (Sigma, A2052), DAPI (Beyotime, C1006), cholera toxin subunit B (CTB)555 (Molecular Probes, Eugene, USA)), Nissl staining solution (Beyotime, C0117), DAB-staining kit (ZLI-9031, ZSGB-BIO), atropine sulfate salt monohydrate (Sigma-Aldrich, A0257) and mecamylamine hydrochloride (Sigma-Aldrich, M9020).

### Stereotactic viral delivery

To investigate the effect of hTau aggregation, pAAV-CAG-Vector/hTau-eGFP virus (serotype: AAV2/8, viral titers: 1.27 × 10^13^ vg/ml 0.8 μl) was injected into the MS of male C57BL/6 mice at the age of 12 weeks. To explore the effect of hTau on excitatory neurons, Cre-inducible recombinant pAAV-EF1α-DIO-hTau-eGFP (serotype: AAV2/8, viral titers:1.38 × 10^13^ vg/ml, 0.8 μl) or pAAV-DIO- EF1α-eGFP- Vector (serotype: AAV2/8, viral titers:1.01 × 10^12^ vg/ml, 0.8 μl) and pAAV-CaMKII-Cre-mCherry (serotype: AAV2/8, viral titers:2.5 × 10^11^ vg/ml, 0.8 μl) were injected into the MS of male C57BL/6 mice at the age of 12 weeks. To explore the effect of hTau on MS cholinergic neurons, AAV-DIO-hTau/Vector-eGFP was injected into the MS of 12-week-old male ChAT-Cre mice. pAAV-EF1α-DIO-ChR2(H134R)-eYFP (serotype: AAV2/8, viral titers:1.01 × 10^13^ vg/ml, 0.8 μl) was injected into the MS of 12-week old male ChAT-Cre mice. In the transsynaptic tracing experiments, H129-G4 was injected into the MS of male C57BL/6 mice at 12 weeks of age. To anterogradely trace the cholinergic MS-HP inputs, pAAV-EF1α-DIO-ChR2(H134R)-eYFP (serotype: AAV2/8, viral titers:1.01 × 10^13^ vg/ml, 0.8 μl) was injected into the MS of 12-week male ChAT-Cre mice. For retrograde tracing, CTB555 was injected into the CA1 and CA3 of 12-week-old male ChAT-Cre-hTau/Vector mice.

### Stereotaxic surgery

Mice were anesthetized with pentobarbital sodium (1%, 30 mg per kilogram) and immobilized in a stereotactic injection apparatus (RWD 68046 & 68055, China). After the scalp was disinfected and balanced, a tiny hole was punched very gently. Viruses were injected into the MS (AP: + 0.86 mm, ML: 0 mm, DV: -4.15 mm), dCA1 (AP: -1.9 mm, ML: ± 1.3 mm, DV: -1.5 mm) and dCA3 (AP: -2.2 mm, ML: ± 2.7 mm, DV: -2.4 mm) by a 10 μl gas-tight miscrosyringe (KF019, China) under a stereotaxic instrument (World Precision Instruments, USA). The injection rate was 50–100 nl/min (for virus injection) or 30 nl/min (for CTB555 injection). After each injection, the needle was left for 5–10 min to avoid virus spread and aspiration. Lincomycin lidocaine gel was evenly applied to the skull, and then absorbable stitches were used to suture the skin. The mice were placed on a heated blanket waiting for wake-up. Finally, the accuracy of the injection site was confirmed by fluorescent expression. Mice with incorrect virus expression locations were excluded.

### Behavioral test

The open-field test (OFT), novel object recognition test (NOR) and elevated plus maze (EPM) were carried out as described previously [17]. The mice were handled at least 3 consecutive days before behavioral tests. For environmental habituation, mice were transferred to the experimental room 2 h before each test.

### Barnes maze test (BMT)

#### Traditional BMT

Twenty-four hours before training, the mice were habituated in the escape target box for 3 min in the dark. During the next 4-day training trials, each mouse underwent 4 trials per day with 20-min intervals. In each trial, the animals were first covered with an opaque chamber for 10 s in the center of the platform and then allowed to explore the platform freely to find the escape target box within 3 min. The exploration traces were automatically recorded by the camera. If the mice found the target within 3 min, they were allowed to stay in the box for 30 s with light off. Otherwise, they were guided to the target box and stayed in it for 30 s. Between each trial, paper towels soaked with 70% ethanol were used to clean the platform and the escape box. During the probe test (day 5), the escape box was removed and the animals were allowed 90 s to search for the platform. Latency to the target hole, number of errors time spent in the target quadrant, percentage of correct pokes and distance moved were measured.

#### Modified BMT (MBM)

The MBM was similar to the one reported by Wahlstrom et al. [18]. Briefly, MBM consisted of a 1-day training (four consecutive trials, 60 s per trial, 1 min interval) and a probe test 2 days later. If the mice could not find the target within 60 s during training, they were guided to the escape box and kept for 30 s. On the third day, the mice were allowed 3 min to explore the platform without the shelter box. Behavior recording software (Chengdu Taimeng Software Co. Ltd) was used to track the animals and analyze the relevant data.

### Immunostaining

The brain tissue was postfixed in 4% PFA at 4 °C for 24 h after sacrifice, and then switched to 25% and 30% sucrose solutions in turn for at least 2 days. Brains were sliced into 30 μm-thick sections by a cryogenic frozen microtome (CM1860, Leica, Germany), and stored in the antifreeze solution (PBS: ethanediol: glycerol = 5:2:3) at -20 °C.

For immunofluorescence staining, the free-floating sections were washed with PBST (PBS with 0.1% Triton-100) and permeabilized with 0.5% Triton-100 PBS for 20–30 min. QuickBlock™ blocking buffer (P0260, Beyotime) was used for blocking. Then, the sections were incubated with primary antibody (1:200 ~ 1:500, P0023A) at 4 °C overnight and incubated with secondary antibodies (Invitrogen, A11055 or A11081 or A-31573) for 1–2 h at 37 °C in the dark (1:500, P0265). Finally, the sections were counterstained with DAPI and mounted with PBS containing 50% glycerol (pH 7.2) for imaging.

For immunohistochemical staining, the brain sections were incubated with 0.3% H_2_O_2_ (in 0.5% Triton-100 PBS) at 37 °C for 20–30 min before blocking to eliminate endogenous peroxidase activity. After incubation with primary antibody, the secondary antibody (AD048/AD049) and ternary antibody (AD050) or hypersensitive ready-to-use goat two-step detection kit (PV-9003, ZSGB-BIO) and DAB-staining kit (ZLI-9031, ZSGB-BIO) were used for horseradish peroxidase reaction staining. Then the brain sections were sealed with neutral balsam after dehydration through a graded ethanol series and hyalinization with dimethylbenzene.

Images were obtained by laser scanning confocal microscopy (LSM780, Zeiss, Germany) and automatic scanning microscopy (VS120, Olympus, Japan). ImageJ was used for image analysis. To estimate optical density in the regions of interest, the photographs were converted into a greyscale and compared to a calibrated greyscale taken from the ImageJ optical density calibration protocol [19].

### Nissl staining

The brain slices were rinsed in 0.1% Triton 100-PBS and mounted on adhesive microscope slides to dry before incubation with Nissl Staining Buffer (C0117, Beyotime, China) for 10 min at 37 °C. Then, the slices were washed in d_2_H_2_O quickly, immersed in 95% ethanol for 5 min, twice, and soaked in 100% ethanol for 5 min for dehydration. Subsequently, the slices were hyalinized in xylene. All slices were covered with diluted neutral gum. The images were acquired with an automatic section scanning system (VS120, Olympus, Japan).

### In vitro electrophysiological recording

After anesthetization with 1% pentobarbital sodium (30 mg per kilogram), mice were sacrificed, and the brains were moved into ice-cold cutting buffer: 225 mM sucrose, 3 mM KCl, 1.25 mM NaH_2_PO_4_, 6 mM MgSO_4_,10 mM glucose, 24 mM NaHCO_3_, and 0.5 mM CaCl_2_ (pH 7.2–7.4; 300 mOsm) saturated with carbogen (95% O_2_ and 5% CO_2_). Brain slices containing the MS (300 μm) were prepared with Vibratome (Campden 7000 smz, UK) and incubated at 34 °C in aCSF saturated with carbogen (95% O_2_ and 5% CO_2_) for 30 min and then incubated at 25 °C in aCSF with 95% O_2_ and 5% CO_2_ for at least 30 min. The aCSF consisted of components including 126 mM NaCl, 3 mM KCl, 1.25 mM NaH_2_PO_4_, 24 mM NaHCO_3_, 2 mM MgSO_4_, 2 mM CaCl_2_, and 10 mM glucose. The slices were transferred into a chamber and maintained with circulating oxygen-saturated aCSF perfusion (1–2 ml/min). The location of MS and the fluorescent expression in the cholinergic neurons were identified under a patch-clamp dedicated fluorescence microscope (BX51WI, Olympus, Japan). Patch pipettes (5–8 MΩ) were pulled from the silicon borate glass electrode (BF150-86-15, Sutter, CA) with a P97 puller (Sutter Instrument, Novato, CA). The whole-cell patch-clamp configuration was employed in voltage-clamp mode (Vm = -65 to -80 mV depending on the resting membrane potential of the cell type). For the current-clamp experiment, the recording pipette were filled with pipette solution: 140 mM K-gluconate, 10 mM KCl, 20 mM HEPES, 3 mM MgCl_2_, 10 mM EGTA, and 1 mM CaCl_2_ (pH adjusted to 7.35 with KOH; 290–300 mOsm, filtered by a 0.22 μm filter).

To stimulate cholinergic neurons with ChR2 expression, light pulses (472 nm, 5 ms in duration) were delivered through the 40 water-immersion objective of an Olympus microscope. An LED illumination resource (DC4100, Thorlabs, US) served as a light source. Ten-ms light pulses were set at 4 Hz, 8 Hz, 10 Hz, 20 Hz and 40 Hz, and were triggered by the corresponding Clamp10 software (Molecular Devices, US). All chemicals were purchased from Sigma. Data recordings were made using a Multiclamp 700B amplifier (Molecular Devices, Sunnyvale, CA). Analogue signals were low-pass filtered at 1 kHz and digitized at 10 kHz using Digidata 1550B and pClamp10 software (Molecular Devices, Sunnyvale, CA). Off-line analysis was performed using Clampfit software (Molecular Devices, Sunnyvale, CA).

### Western blotting

Western blotting was performed as described in a previous study [17]. To dissect out the MS region, we first used a vibratome (Campden 7000 smz, UK) to slice the mouse brains in ice-cold artificial cerebrospinal fluid (aCSF). Then, brain slices from AP + 1.1 mm to AP + 0.5 mm were collected. A 0.5 mm X 1.0 mm tissue per slice was manually dissected at the intersection of the midline and the base of the lateral ventricle. Finally, the dissected brain tissues were homogenized with RIPA buffer (Beyotime) for western blotting. Proteins were separated in 10% SDS-PAGE gels and transferred onto nitrocellulose membranes (Whatman). The membranes were then blocked and incubated with primary antibody and secondary antibodies (1:500 – 1:2000 dilutions). Blots were visualized using an ECL system (ChemiScope 6000, Shanghai, China).

### Photoactivation experiments

Mice were anesthetized with isoflurane (5% and 400 ml/min for induction; 2% and 100–150 ml/min for maintenance) delivered by Matrx VMR (MIDMARK, US). After checking transmittance (Throlabs, US), sterilized optical fibers (200 μm O.D., 0.37 mm numerical aperture (NA); inper, Hangzhou, China) were planted into the MS (AP: + 0.86 mm, ML: 0 mm, DV: -4.15 mm) and dCA1 (AP: -1.9 mm, ML: ± 1.3 mm, DV: -1.5 mm) of mice with ChR2 expression. During photostimulation, 473 nm blue light (20 Hz, 10 ms pulse wave, 90 s duration, or 15 min of 2 s trains at different frequencies, 5 ms pulse duration) was delivered. To determine whether the effects of light stimulation were due to delayed effects on behavior during the memory retention test and to identify the time-limited nature of memory consolidation, a 3 h delay experiment was conducted in which mice received optical stimulation 3 h after the last training trial in the MBM. To block the AChRs simultaneously with light stimulation, cannulas (Catalog No.62034, No.62134, No.62524, RWD Life Science, China) were implanted into the bilateral hippocampal CA1 (AP ± 1.9 mm, ML ± 1.3 mm, DV-1.5 mm), and a blocker cocktail (0.8 μl) containing atropine sulfate monohydrate (7.2 mM, Sigma-Aldrich, A0257) and mecamylamine hydrochloride (10 mM, Sigma-Aldrich, M9020) was injected through the cannulas 30 min before light stimulations [18, 20].

### In vivo electrophysiological recording

Mice were anesthetized with isoflurane (5% isoflurane, 400 ml/min for induction; 2% isoflurane, 100–150 ml/min for maintenance) through Matrx VMR (MIDMARK, US). Then, customer-designed tetrodes with tungsten electrodes were implanted in the brains, in which 6-channel electrodes were used for recording in the MS (AP: + 0.86 mm, ML:0 mm, DV: -4.15 mm) and 8-channel electrodes were used for recording in the dCA1 (AP: -1.9 mm, ML: -1.3 mm, DV: -1.5 mm). Next, the mice were placed on an electric blanket for recovery. After tetrode implantation, mice were individually housed. Each recording lasted 20 min and was made at 3 different time points, i.e., 1 day before the MBM training, within 3 h after the last trial of MBM and 4 days after MBM. All electrophysiological signals were recorded by the OmniPlex D Neural Data Acquisition System (Plexon, Hongkong, China) and analyzed off-line [21, 22]. Signals were filtered at 0.05–8000 Hz and amplified at a gain of 500. For spectral analysis, the LFP signals (0–4 Hz, 4–8 Hz, 8–13 Hz, 13–30 Hz, 30–50 Hz, 50–100 Hz) were analyzed by MATLAB (MathWorks) and Chronux, an open-source software package for the analysis of neural data. For coherence analysis, the paired signals from MS and dCA1 were normalized by cross-spectral density using Neuro Explore automatic methods. The coherence value (0–1) is a measurement of the interdependence of two signals in frequency domains. A value of 0 means that the two signals are independent at the considered frequency, whereas a value of 1 means that the signals are identical in frequency and have a constant phase relationship. Enhancement of coherence signifies an increase in frequency similarity and phase consistency between oscillatory signals from two brain regions. Averaged coherence was obtained by averaging the values in the above bands.

### Statistical analysis

GraphPad Prism (version 8.0) was used for statistical analyses. Student’s t test was used for two-group comparisons. One-way ANOVA or two-way ANOVA followed by post hoc tests was conducted for multiple comparisons. Animals with incorrect virus injections, incorrect fibers and cannula placement were excluded from the analysis. All data were presented as mean ± SEM. *P* < 0.05 was considered as statistically significant.

## Results

### Overexpressing hTau in the MS induced spatial memory loss with cholinergic degeneration in a time-dependent manner

**Fig. 1:**
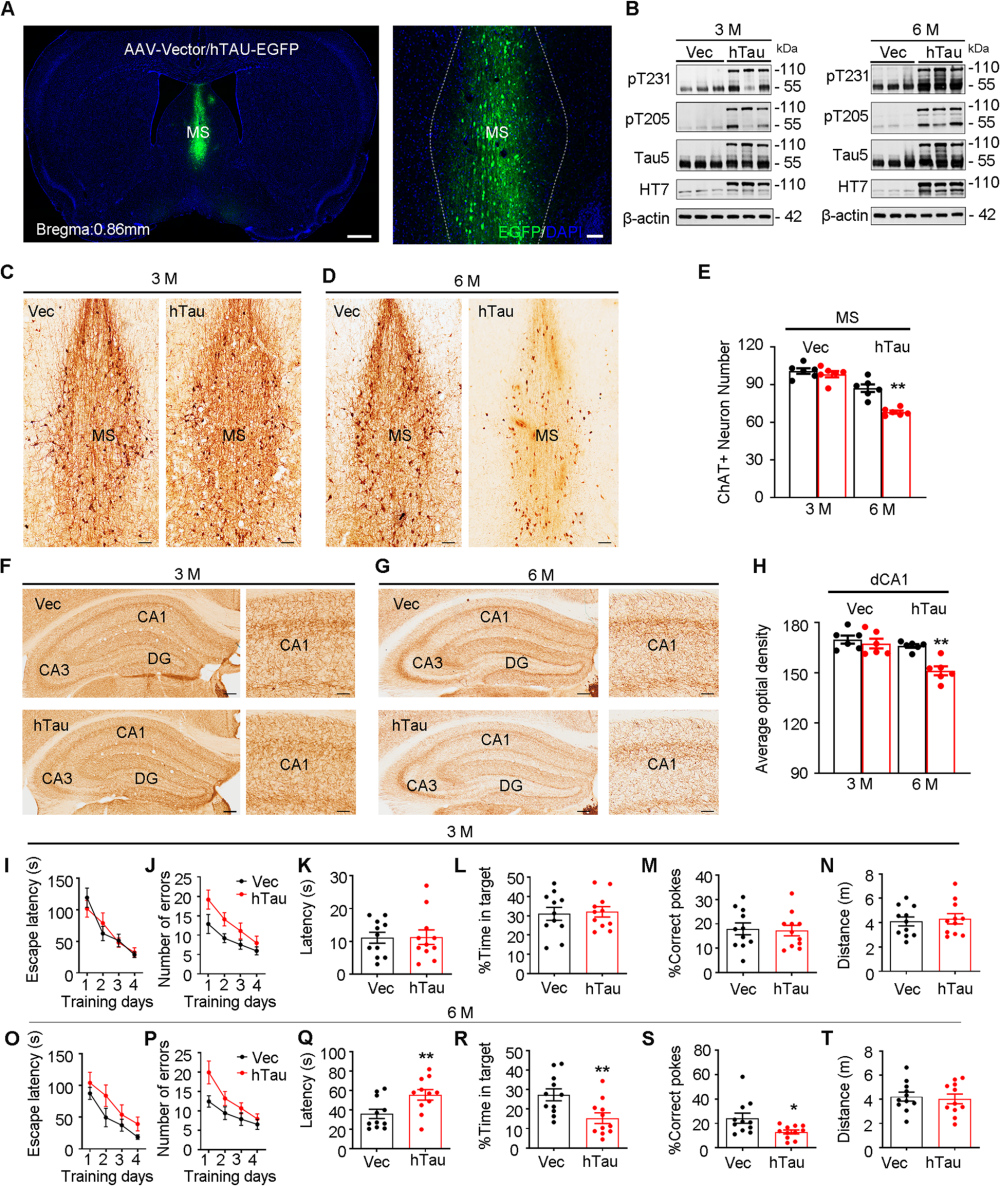
Overexpressing hTau in MS induces MS-dCA1 cholinergic loss and spatial memory deficit. **A** The exogenously overexpressed hTau detected by confocal imaging (**A**) and Western blotting (**B**) at 3 and 6 m after MS infusion of pAAV-CAG-MAPT-EGFP-3flag or pAAV-CAG-EGFP-3flag, respectively. The antibody pT231, pT205, Tau5 and HT7 reacts with phosphorylated tau and total tau proteins. The bands at 110 kDa and 55 kDa indicate exogenously expressed hTau and endogenously expressed mouse tau respectively. **C**-**E** Overexpressing hTau in the MS for 6 m but not 3 m significantly reduced cholinergic neuron number measured by ChAT immunohistochemical staining. *N* = 6 mice per group. ChAT + cell number: unpaired *t* test, t = 0.7513 df = 10, *P* > 0.05 (3 m); t = 5.802 df = 10, *P* < 0.01 (6 m). Scale bar, 100 μm. **F**–**H** Overexpressing hTau for 6 m but not 3 m decreased cholinergic projection intensity in hippocampal CA1 measured by ChAT immunohistochemical staining. *N* = 6 mice per group. ChAT + fiber intensity (**H**): unpaired *t* test, t = 0.6429 df = 10,* P* > 0.05 (3 m); t = 5.178 df = 10, *P* < 0.01 (6 m). Scale bar, 200 μm or 50 μm in enlarged image. **I**-**N** Overexpressing hTau in the MS for 3 m had no effects on spatial learning (**I**, **J**) and memory (**K**-**N**) detected by Barnes maze (BM) test. **I** two–way ANOVA group × days, escape latency: F [3,80] = 0.7710, *P* > 0.05; **J** Number of errors: F [3,80] = 0.5044, *P* > 0.05; **K** first escape latency, unpaired *t* test, t = 0.06583 df = 20, *P* > 0.05; **L** percentage of time in target, unpaired *t* test, t = 0.2421 df = 20, *P* > 0.05; **M** percentage of correct pokes, unpaired *t* test, t = 0.2261 df = 20, *P* > 0.05; **N** distance moved, unpaired *t* test, t = 0.3920 df = 20, *P* > 0.05). *N* = 11 mice per group. **O**-**T** Overexpressing hTau in MS for 6 m impaired spatial memory (**Q**-**T**) without changing learning ability (**O**, **P**) in BM test. **O** Two–way ANOVA group × days, escape latency: F [3,80] = 0.2125, *P* > 0.05; **P** two–way ANOVA group × days, number of errors: F [3,80] = 1.124, *P* > 0.05; **Q** unpaired *t* test, t = 2.714 df = 20, *P* < 0.05; **R** percentage of time in target, unpaired *t* test, t = 2.906 df = 20, *P* < 0.01; **S** percentage of correct pokes, unpaired *t* test, t = 2.530 df = 20, *P* < 0.05; **T** distance moved, unpaired *t* test, t = 0.3526 df = 20, *P* > 0.05. *P < 0.05, **P < 0.01. N = 11 mice per group, and data were presented as mean ± SEM

Postmortem studies have shown abundant tau pathology in the basal forebrain of AD patients [23]. As MS is one of the most vulnerable subregions of the basal forebrain in AD [24], we first measured phosphorylated tau in 9-m 3 × Tg and 5 × FAD AD mice, two widely used AD mouse models. Compared with the age-matched wild-type controls, a significantly increased accumulation of hyperphosphorylated tau (at Thr231 and Thr205) was detected in the MS subset of the AD mice (sFig. 1), which confirmed the vulnerability of MS to tau pathology. Then, we investigated the causal role of tau accumulation in MS lesions by stereotaxically infusing pAAV-CAG-hTau-eGFP (serotype: AAV2/8, viral titers: 1.27 × 10^13^ vg/ml, 0.8 μl) into the MS of C57BL/6 mice (MS-hTau mice). After 3 or 6 m, robust overexpression of hTau in the MS was confirmed by immunofluorescence imaging (Fig. 1A) and western blotting using Tau5, an antibody that reacts with total tau proteins (Fig. 1B). Given that MS is enriched in cholinergic neurons, we employed ChAT staining, a marker for cholinergic neurons, to evaluate cholinergic lesions in the presence of tau pathology. By counting ChAT + neurons and measuring their projecting intensity, a significant loss of cholinergic neurons in the MS with a prominent reduction of cholinergic fibers in both MS and hippocampus was shown at 6 m but not 3 m after pAAV-CAG- hTau-eGFP infusion, when compared with the empty vector control groups (Fig. 1C-H).

To evaluate spatial cognitive capacity, we then conducted the Barnes maze (BM) test. The performance of MS-hTau mice was identical to that of the controls during 4 days of training (Fig. 1I, J, O, P) at both 3 m and 6 m after virus injection, indicating intact spatial learning ability in the presence of MS-hTau accumulation. In the probe trial, the MS-hTau mice showed spatial memory deficits at 6 m but not at 3 m after pAAV-CAG- hTau-eGFP injection, as evidenced by longer escape latency, less time in the target quadrant and fewer correct pokes than the control mice (Fig. 1K-N, Q-T).

These data together demonstrate that hTau accumulation in the MS induces cholinergic degeneration and impairs hippocampus-dependent spatial memory in a time-dependent manner.

### Cholinergic hTau accumulation triggers cholinergic impairments in both MS and hippocampus with spatial memory deficits

The MS region of the basal forebrain contains cholinergic neurons, glutamate neurons and GABA neurons [25]. By double immunofluorescence staining, we confirmed that ~ 94% of cholinergic neurons were CaMKII+, a marker for excitatory neurons, and ~ 28% of cholinergic neurons were GABA + , a marker of inhibitory neurons, under physiological conditions (sFig. 2A, C). Further analysis data showed that 13.3% of CaMKII + and 7.1% of GABA + neurons were ChAT + (sFig. 2B, D). Then, we investigated whether overexpressing hTau in CaMKII+ neurons could mimic hTau-impaired spatial memory. By stereotaxically injecting pAAV-CaMKII-Cre-mCherry and pAAV-EF1a-DIO-hTau-eGFP (1:1) into the MS of C57BL/6 mice (MS-CaMKII-hTau mice), we observed that ~ 94.6% of hTau was targeted in CaMKII+ neurons (sFig. 3A, B), which confirmed the efficiency of the Cre-LoxP system. In the BM test, we found no difference in spatial learning (sFig. 3C, D, I, J) and memory (sFig. 3E-H, K-M) between MS-CaMKII-hTau mice and the controls at both 3 m and 6 m after virus injection. Moreover, no anxiety-like behaviours were detected in MS-CaMKII-hTau mice, as evidenced by the unchanged open arm entries (sFig. 3O, S) and staying time in the open arm during the elevated plus maze test (sFig. 3P, T) and the unchanged time in the centre zone during the open field test (sFig. 3Q, U). No motor dysfunction was detected in either group (sFig. 3H, N, R, V). Therefore, overexpressing hTau in CaMKII+ neurons in the MS could not phenotype MS tau accumulation-induced spatial memory loss.

**Fig. 2:**
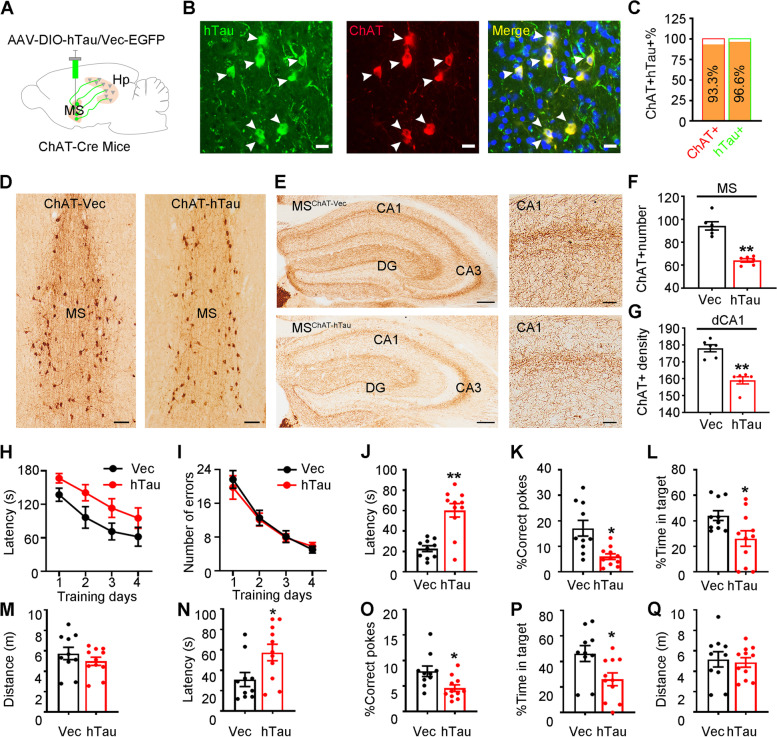
Overexpressing hTau in ChAT-neurons induces spatial memory deficit and cholinergic loss. **A** Carton shown infusion of AAV-DIO-hTau-EGFP or the empty vector control in the MS of ChAT-Cre mice. **B**, **C** Representative co-immunofluorescence images confirmed cholinergic-specific expression of the exogenously infused hTau in the MS (**B**), and quantitative analysis for the percentage of ChAT +- and hTau +- neurons (**C**). Scale bar, 15 μm. *N* = 6 mice. **D**-**G** Overexpressing hTau for 3 m in ChAT + -neurons caused cholinergic neuron loss (**D** scale bar, 100 μm) in the MS and in its cholinergic projection to hippocampal CA1 (**E** scale bar, 200 μm or 50 μm in enlarged images) measured by ChAT immunohistochemical staining. *N* = 6 mice per group. ChAT + cell number (**F** unpaired t test, t = 7.686 df = 10, *P* < 0.01). ChAT + fiber intensity (**G** unpaired t test, t = 6.382 df = 10, *P* < 0.01). **P < 0.01 vs Vec group. **H**-**Q** MS-ChAT-hTau mice displayed long-term spatial memory deficits measured by BM test. During spatial learning trials, no difference of latency (**H** two–way ANOVA group × days, escape latency: F [3,76] = 0.1017, *P* > 0.05) and error number (**I** two–way ANOVA group × days, number of errors: F [3,76] = 0.2126, *P* > 0.05) were found between MS-ChAT-hTau mice and the controls. In probe test carried out 24 h after the last training trial (**J**-**L**), MS-ChAT-hTau mice showed longer time to reach the target area (**J** unpaired t test, t = 5.063, df = 19, *P* < 0.01), with less poking to the target hole (**K** unpaired t test, t = 3.522 df = 19, *P* < 0.01) and less staying time in the target area (**L** unpaired t test, t = 2.439 df = 19, *P* < 0.05) than the controls. After one week (**N**-**P**), the MS-ChAT-hTau mice still showed an increased latency to the target (**N** unpaired t test, t = 2.483, df = 19, *P* < 0.05) with decreased correct pokes (**O** unpaired t test, t = 2.763 df = 19, *P* < 0.05) and time staying in the target (**P** unpaired t test, t = 2.556 df = 19, *P* < 0.05) compared with control mice. No difference of distance moved was shown between groups (**M** unpaired t test, t = 1.061 df = 19, *P* > 0.05; **Q** unpaired t test, t = 0.3439 df = 19, *P* > 0.05). *N* = 10, 11 mice per group. *P < 0.05, **P < 0.01 vs Vec group. Data were presented as mean ± SEM

Then, we targeted cholinergic neurons specifically by injecting pAAV-EF1α-DIO-hTau-eGFP into the MS of ChAT-Cre mice (Fig. 2A). After 3 m, we observed that 98.2% of hTau-eGFP immunoreactivity was colocalized with ChAT (Fig. 2B, C), which confirmed the successful overexpression of hTau in cholinergic neurons with the Cre-LoxP system (MS-ChAT-hTau mice). To evaluate the impact of cholinergic hTau accumulation on cholinergic lesions, we performed quantitative analysis on ChAT+ neurons and their fibers. Compared with the control group, the number of ChAT+ neurons in the MS was significantly decreased in MS-ChAT-hTau mice (Fig. 2D, F). In the hippocampus, the intensity of cholinergic fibers was much weaker in the MS-ChAT-hTau group than in the controls (Fig. 2E-G). These data demonstrate that cholinergic hTau accumulation not only induces cholinergic neuron loss but also impairs cholinergic projections from the MS to the hippocampus.

In the BM test, MS-ChAT-hTau mice exhibited comparable latencies and error times to reach the correct locations during the 4-day training trials compared to the controls (Fig. 2H, I), indicating that hTau accumulation did not affect the spatial learning of the mice. In the probe test performed at 24 h and one week after the last training, the MS-ChAT-hTau mice showed spatial memory deficits, as evidenced by the significantly increased time to reach the target and decreased number of correct pokes compared with the control mice (Fig. 2J-L, N-P). No motor dysfunction was detected, as evidenced by the identical distance moved during the BM test (Fig. 2M, Q).

**Fig. 3:**
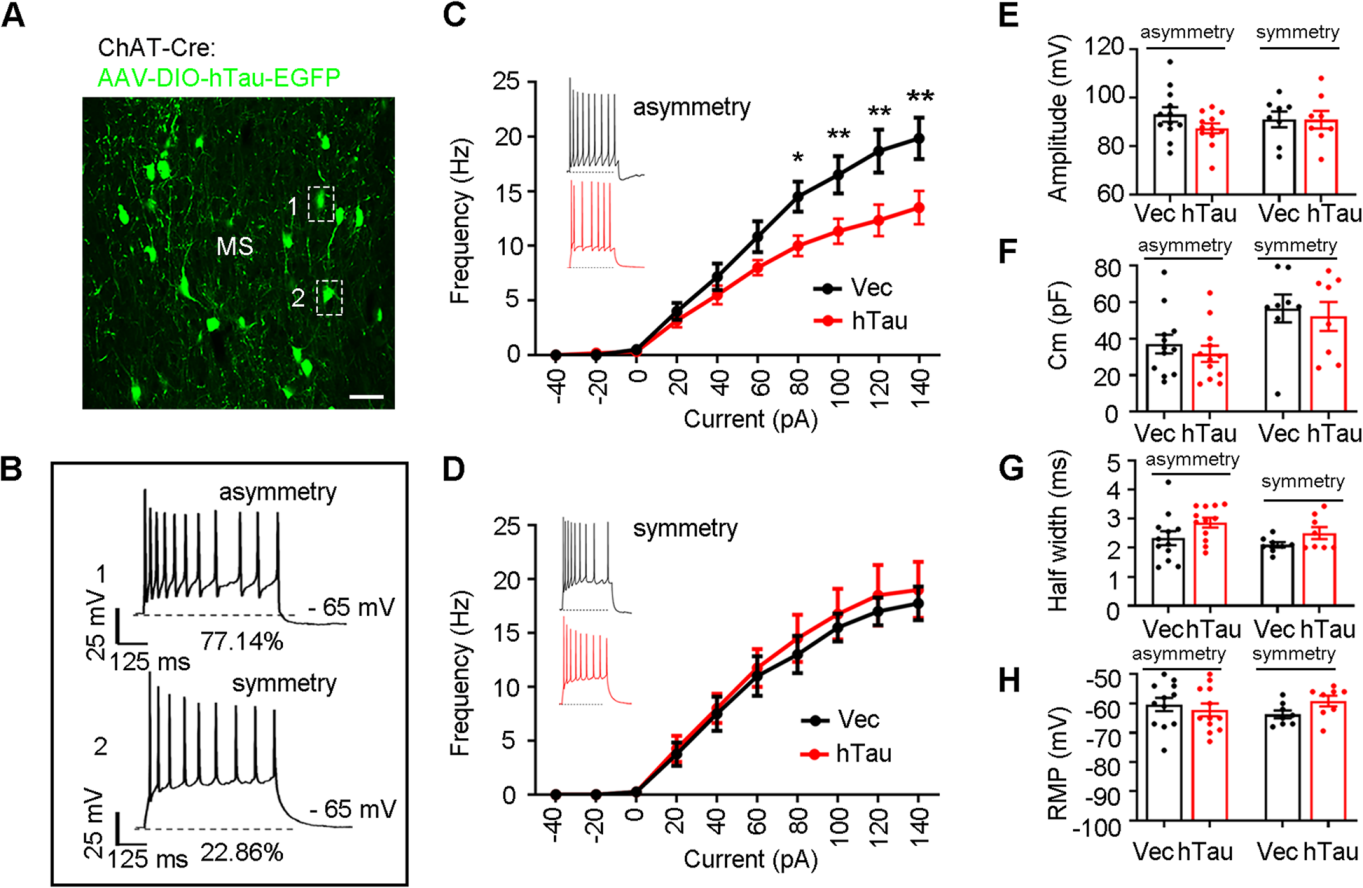
Cholinergic accumulation of hTau inhibits asymmetrical discharges with no influence on the symmetrical currents. **A** Representative image showing hTau accumulation in cholinergic neurons. Scale bar, 50 μm. **B** Two discharge waveforms recorded in cholinergic neurons: asymmetric (top) and symmetrical (bottom). The baseline membrane potential is -65 mV. **C**, **D** MS-ChAT-hTau mice showed a significantly decreased excitability in asymmetrically discharged cholinergic neurons (**C** two–way ANOVA group × current, frequency: F [9,220] = 2.758, *P* < 0.01) with no significant changes in the symmetrically discharged cholinergic neurons (**D** two–way ANOVA group × current, frequency: F [9,140] = 0.07977, *P* > 0.05). **E**–**H** No differences in amplitude (**E** asymmetrically, unpaired *t* test, t = 1.596 df = 22, *P* > 0.05; symmetrically, unpaired t test, t = 0.02285 df = 14, *P* > 0.05), CM (**F** asymmetrically, unpaired *t* test, t = 0.7843 df = 22, *P* > 0.05; symmetrically, unpaired t test, t = 0.3898 df = 14, *P* > 0.05), half-width (**G** asymmetrically, unpaired *t* test, t = 1.832 df = 22, *P* > 0.05; symmetrically, unpaired *t* test, t = 1.785 df = 14, *P* > 0.05), and RMP (**H** asymmetrically, unpaired *t* test, t = 0.5846 df = 22, *P* > 0.05; symmetrically, unpaired t test, t = 2.002 df = 14, *P* > 0.05) were detected between MS-ChAT-hTau group and the controls in neither asymmetrically- nor symmetrically- discharged cholinergic neurons in the MS. *N* = 5 mice per group (12 recordings in asymmetrically discharged cholinergic neuron and 8 recordings in symmetrically discharged cholinergic neuron). *P < 0.05, **P < 0.01 vs control group

By ex vivo brain slice recording (Fig. 3A), we identified two distinct types of MS cholinergic neurons under physiological conditions: one had symmetrical discharge characteristics (22.86%), while the other showed asymmetric discharge characteristics (77.14%) (Fig. 3B). Interestingly, hTau accumulation specifically inhibited the excitability of the asymmetric discharge neurons (Fig. 3C, D) without changing the amplitude, membrane capacitance (Cm), halfwidth of action potential and resting membrane potential (RMP, Fig. 3E-H). These data together demonstrate that the cholinergic accumulation of hTau induces hippocampus-dependent spatial memory deficits with the dysfunction of cholinergic neurons in MS.

### Activating cholinergic neurons in the MS during memory consolidation rescues hTau-induced spatial memory decline

**Fig. 4:**
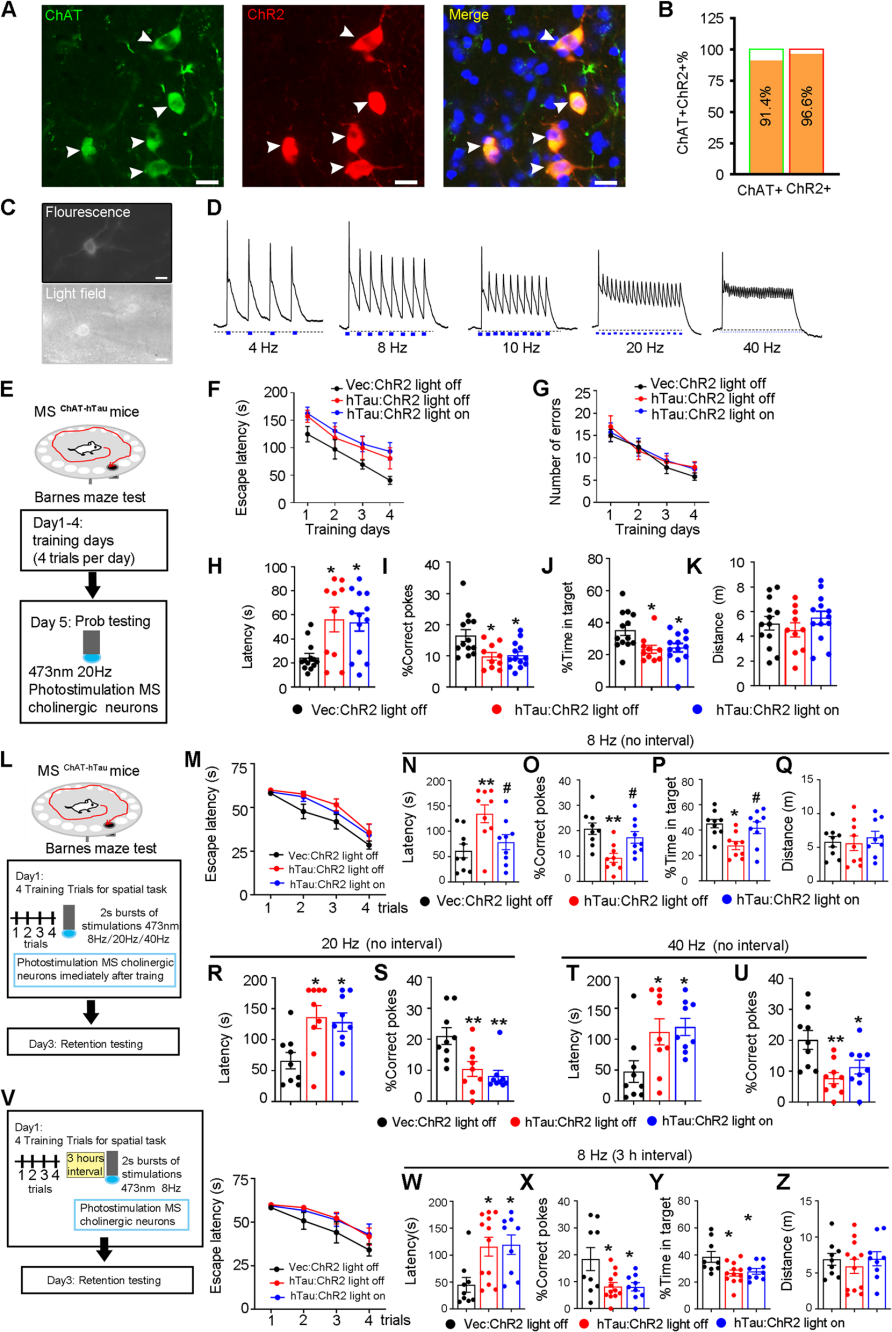
Photoactivating cholinergic neuron at theta-rhythm within 3 h- consolidation time window efficiently rescues hTau-induced spatial memory deficit. **A**, **B** Cholinergic-specific expression of ChR2 by Cre-LoxP strategy, and ~ 97% ChR2 + neurons were ChAT + measured by immunofluorescence staining of ChAT. *N* = 6 mice per group. Scale bar, 20 μm. **C**, **D** Cholinergic neurons expressing ChR2 fired upon 473 nm blue light stimulation at different frequencies. *N* = 3 mice per group. Scale bar, 20 μm. **E** Diagram shown the traditional BM test. We photostimulated cholinergic neurons (473 nm, 20 Hz) during probe test at day 5. **F**-**K** MS-ChAT-hTau mice showed comparable spatial learning ability to controls during 4-day learning trials (two–way ANOVA group × days, **F** escape latency: F [6,132] = 0.1181, *P* > 0.05; **G** number of errors: F [6,132] = 0.2038, *P* > 0.05). Photoactivation of cholinergic neurons at 20 Hz could not reverse the increase of latency (**H** One–way ANOVA group, F [2, 33] = 6.379, *P* < 0.01), decrease of %correct poke (**I** One–way ANOVA group, F [2, 33] = 6.341, *P* < 0.01) and reduction of %time in target (**J** One–way ANOVA group, F [2, 33] = 5.296, *P* < 0.05) in hTau-ChR2-light off group. No difference of distance moved was among groups (**K** One–way ANOVA group, F [2, 33] = 0.7610, *P* > 0.05). *P < 0.05 vs Vec: ChR2 light off. *N* = 10–13 mice per group. **L** Diagram shown the modified BM test (MBM): mice take 4 training trials for spatial task in day 1 and a test on day 3. Photostimulating cholinergic neurons for 15 min was conducted after the forth training trial immediately. **M**-**Q** Photoactivating cholinergic neurons at 8 Hz during memory consolidation rescued spatial memory loss in MS-ChAT-hTau mice. During spatial training, escape latency of MS-ChAT-hTau group was not different from controls (**M** two–way ANOVA group × days, escape latency: F [6,96] = 0.3494, *P* > 0.05). In memory test, photostimulating at 8 Hz reversed the increased latency (**N** One–way ANOVA group, F [2, 24] = 6.16, *P* < 0.01), the decreased correct poke (**O** One–way ANOVA group, F [2, 24] = 7.793, *P* < 0.01) and the reduced time in target (**P** One–way ANOVA group, F [2, 24] = 5.85, *P* < 0.01) in hTau-ChR2-light off group. *P < 0.05, **P < 0.01 vs Vec: ChR2 light off; ^#^*P* < 0.05 vs hTau: ChR2 light off. *N* = 9 mice per group. **R**-**U** Photoactivating cholinergic neurons at 20 Hz or 40 Hz immediately after training on day 1 in MBM could not reverse spatial memory impairment in MS-ChAT-hTau mice. No differences in latency to reach the target area (**R** 20 Hz, One–way ANOVA group, F [2, 24] = 6.045, *P* < 0.01; **T** 40 Hz, One–way ANOVA group, F [2, 24] = 4.984, *P* < 0.01) and %correct poke (**S** 20 Hz, One–way ANOVA group, F [2, 24] = 8.682, *P* < 0.01; **U** 40 Hz, One–way ANOVA group, F [2, 24] = 6.877, *P* < 0.01) were found between light on and light off groups. *P < 0.05, **P < 0.01 vs Vec: ChR2 light off. *N* = 9 mice per group. **V**-**Z** At 3 h after last training, photoactivating cholinergic neurons no longer improved hTau-induced spatial memory deficits (**V** light panel, two–way ANOVA group × days, escape latency: F (6,108) = 0.5270, *P* > 0.05; **W** One–way ANOVA group, F [2, 27] = 5.788, *P* < 0.01; **X** One–way ANOVA group, F [2, 27] = 5.001, *P* < 0.01; **Y** One–way ANOVA group, F [2, 27] = 5.252, *P* < 0.01). There were no differences in distance between light on and light off groups (**Q** One–way ANOVA group, F [2, 33] = 0.7610, *P* > 0.05; **Z** One–way ANOVA group, F [2, 27] = 0.4052, *P* > 0.05). *P < 0.05 vs Vec: ChR2 light off; ^#^*P* < 0.05 vs hTau: ChR2 light off. *N* = 9–12 mice per group. Data were presented as mean ± SEM

To explore whether photostimulating cholinergic neurons in the MS could rescue hTau-induced spatial memory deficits, we selectively expressed ChR2 in cholinergic neurons by infusing AAV-EF1a-DIO-hChR2-mCherry into the MS of ChAT-Cre mice (*ChAT:: ChR2* mice). The targeting specificity was confirmed by immunofluorescence staining of ChAT in the MS (Fig. 4A, B). By whole-cell patch clamp recording, we found that blue light stimulation at 4 Hz, 8 Hz, 10 Hz, 20 Hz, and 40 Hz induced corresponding firings of action potentials in ChR2-expressing cholinergic neurons (Fig. 4C, D), which confirmed high temporal precision activation of cholinergic neurons in the MS by blue light.

Given that only memory deficits but not learning impairment were detected in MS-ChAT-hTau mice, we speculate that activating cholinergic neurons in the probe trial may be able to rescue the memory dysfunction induced by cholinergic hTau accumulation (Fig. 4E). Unexpectedly, we did not observe a significant difference between the light-on and light-off groups of MS-ChAT-hTau mice, in which the latency to the target and incorrect pokes were significantly greater than those of the MS-ChAT-eGFP control mice (Fig. 4F-K). These data indicate that photoactivating cholinergic neurons in the MS during the memory retrieval phase cannot improve spatial memory impaired by cholinergic hTau accumulation.

In addition to information retrieval, memory capacity is highly determined by the consolidation phase [26, 27]. To investigate whether and how cholinergic neurons with hTau accumulation may drive consolidation dysfunction thereby leading to memory deficits, we modified the traditional Barnes maze paradigm (MBM) and photoactivated cholinergic neurons in the MS immediately after a 1-day training trial (Fig. 4L). Notably, photostimulating at 8 Hz (Fig. 4N-Q), but not at 20 Hz or 40 Hz (Fig. 4R-U), remarkably decreased the first latency to the target and increased the number of correct pokes in the MS-ChAT-hTau group, suggesting a rhythm-dependent improvement in hTau-induced spatial memory deficit via memory consolidation. No motor dysfunction was detected, as evidenced by the identical distance moved by all groups in the MBM test (Fig. 4Q).

To further verify the precise efficient time window for memory consolidation, we conducted a 3 h delay blue light activation experiment in the MBM test, in which the MS-ChAT-hTau mice received 8 Hz stimulation for 15 min at 3 h after the last spatial training trial and underwent a memory test 48 h later (Fig. 4V). Compared with the MS-ChAT-hTau mice in the light-off group, MS-ChAT-hTau mice in the light-on displayed similar latencies to the target and correct poke times (Fig. 4W-Z), which further defined a sensitive time window of the spatial memory consolidation. These data suggest that activating cholinergic neurons in the MS could efficiently rescue hTau-induced memory consolidation deficits in a strict theta rhythm- and time window-dependent manner.

### Activating the MS-CA1 cholinergic circuit improves spatial memory impaired by cholinergic tau accumulation

**Fig. 5:**
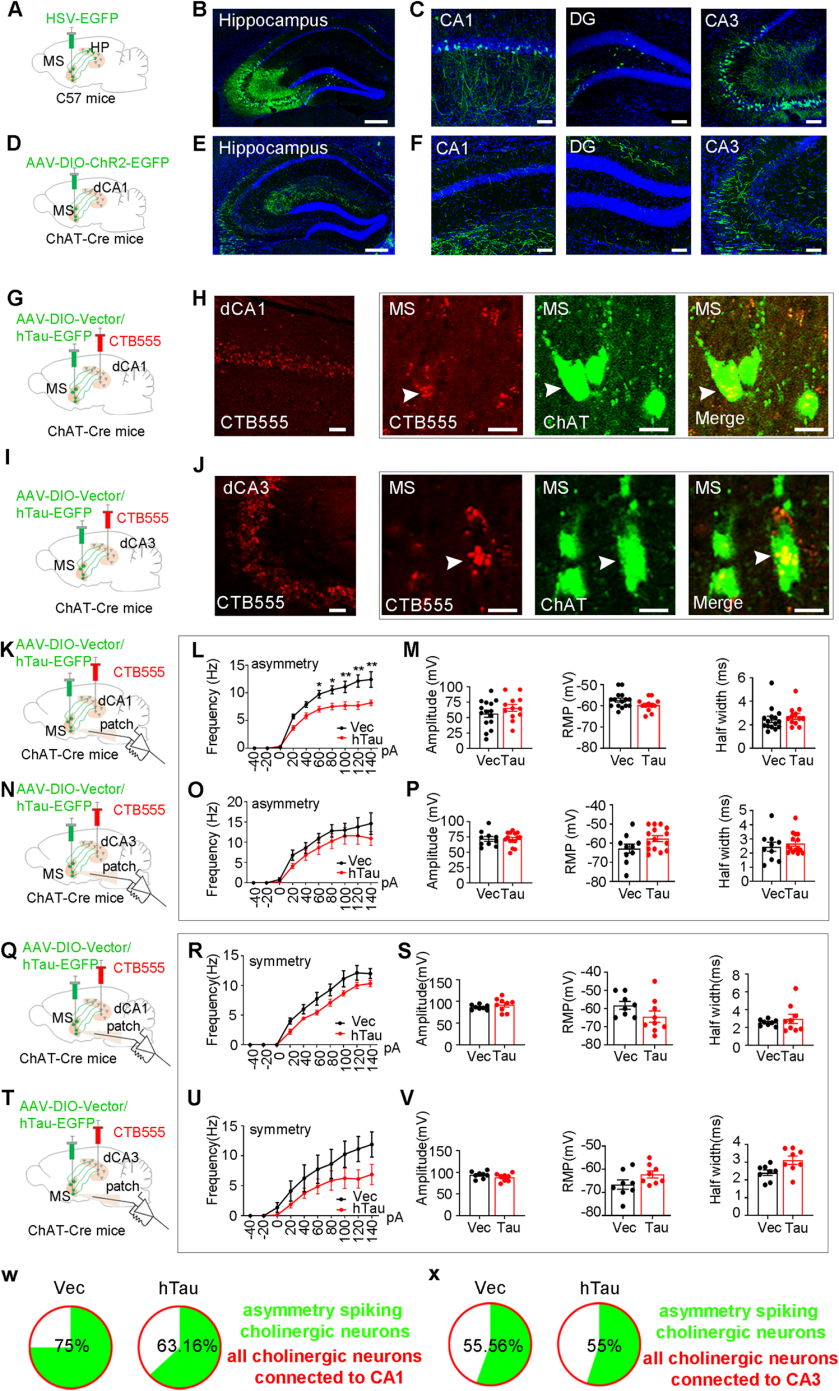
Cholinergic Tau accumulation inhibits MS-CA1 but not MS-CA3 cholinergic pathway. **A**-**C** Anterograde tracing MS-hippocampus circuit in C57BL/6 mice. The trans-synaptic H129-G4 virus was infused into the MS, after 2 days, EGFP expression was detected in the whole hippocampus (**B** Scale bar, 200 μm), including CA1, DG, CA3 (**C** Scale bar, 50 μm). **D**-**F** Anterograde tracing MS-hippocampus circuit in ChAT-Cre mice. AAV-DIO-ChR2-EGFP was infused into the MS for 3 weeks and EGFP + fibers were detected in the hippocampus (**E** Scale bar, 200 μm), especially in CA1, DG, CA3 subregions (**F** Scale bar, 50 μm). *N* = 3 mice per group. **G**, **I** Diagram for conducting cholinergic overexpression of hTau and retrograde tracing of MS-HP circuit. AAV-DIO-hTau was infused into the MS of ChAT-Cre mice to specifically overexpress hTau in cholinergic neurons, and CTB555 was infused in the hippocampal CA1 and CA3 respectively to retrogradely trace MS-CA1 and MS-CA3 circuit. **H**, **J** Fluorescence confirmation of CTB555 expression in the CA1 and CA3 and co-localization of retrograde CTB555 with ChAT + neurons in the MS. **K**, **N**, **Q**, **T** Diagram for patch recording of cholinergic neuron overexpression of hTau in MS of MS-HP circuit especially by retrograde tracing. **K**-**M** Overexpressing hTau significantly decreased the excitability of asymmetrically discharged cholinergic neurons (**L** frequency, two-way ANOVA group × current, F [9,250] = 3.336, *P* < 0.01) in MS-CA1 pathway without changing the amplitude (**M** unpaired t test, t = 1.154, df = 25, *P* > 0.05), RMP (**M** unpaired t test, t = 1.751, df = 25, *P* > 0.05) and half-width (**M** unpaired t test, t = 0.7100, df = 25, *P* > 0.05). **N**-**P** No significant differences in the excitability (**O** two–way ANOVA group × current, F [9,230] = 0.3313, *P* > 0.05), amplitude (**P** unpaired t test, t = 0.1154, df = 25, *P* > 0.05), RMP (**P** unpaired t test, t = 1.960, df = 23, *P* > 0.05) and half-width (**R** unpaired t test, t = 0.6674 df = 23, *P* > 0.05) were detected on the asymmetrically discharged cholinergic neurons in the MS-CA3 pathway between the two group. **Q**–**V** No significant differences were detected in symmetrically discharged cholinergic neurons after overexpressing hTau in MS-CA1 pathway (**R** frequency, two-way ANOVA group × current, F [9,150] = 1.265, *P* > 0.05) without changing the amplitude (**S** unpaired t test, t = 0.9928, df = 15, *P* > 0.05), RMP (**S** unpaired t test, t = 1.589, df = 15, *P* > 0.05) and half-width (**S** unpaired t test, t = 0.7321, df = 15, *P* > 0.05) and MS-CA3 group (**U** two–way ANOVA group × current, F [9,140] = 0.6888, *P* > 0.05), without changing amplitude (**V** unpaired t test, t = 1.358, df = 14, *P* > 0.05), RMP (**P** unpaired t test, t = 1.742, df = 14, *P* > 0.05) and half-width (**R** unpaired t test, t = 0.9857 df = 14, *P* > 0.05). **W**-**X** Quantitative analyses of the asymmetric cholinergic neurons in MS-CA1 and MS-CA3 pathways in ChAT-hTau and control groups. *N* = 6 mice per group. *P < 0.05, **P < 0.01 vs control group. Data were presented as mean ± SEM

The hippocampus is one of the main targets of cholinergic efferents [28] and is primarily associated with spatial memory [29, 30]. First, we performed transsynaptic anterograde tracing to map the potential structural connectivity from cholinergic neurons in the MS to hippocampal neurons. H129-G4 was injected into the MS of C57BL/6 mice. Forty-eight hours later, we observed robust GFP expression in the CA1, CA3 and DG of the dorsal hippocampus (Fig. 5A-C). To verify the monosynaptic cholinergic projection from the MS to CA1, we injected pAAV-EF1α-DIO-ChR2(H134R)-eYFP virus into the MS area of ChAT-Cre mice. Three weeks after injection, the GFP signal was detected in the dorsal hippocampal neuronal fibers, and the fiber density in the CA1 and CA3 was stronger than that in the DG (Fig. 5D-F). These results confirm the structural connection from the MS to the hippocampus under physiological conditions.

Then, we asked whether and which MS-HP subcircuit is vulnerable to tau accumulation. We injected a retrograde tracker (CTB, cholera toxin subunit B) into the CA1 and CA3 of MS-ChAT-hTau mice to label neurons in the MS-CA1 and MS-CA3 circuits, respectively (Fig. 5G-J). By in vitro whole-cell patch-clamp recording of cholinergic neurons in the MS that were colocalized with CTB555 from CA1 or CA3 (Fig. 5K-V), we found that hTau accumulation specifically inhibited the excitability of cholinergic neurons with asymmetric firing patterns in the MS-CA1 circuit (Fig. 5L) but not the MS-CA3 circuit (Fig. 5O), while the amplitude, half width and RMP were unchanged (Fig. 5M, P). No difference in cholinergic neurons with symmetric firing patterns was detected between the vector and hTau group (Fig. 5Q-V). By analyzing the firing characteristics of all cholinergic neurons with circuit connections to CA1 or CA3, we found that overexpressing hTau specifically reduced the proportion of cholinergic neurons connected to CA1 but not to CA3 (Fig. 5W-X). These data indicate that hTau accumulation preferentially inhibits the MS-CA1 cholinergic pathway.

**Fig. 6:**
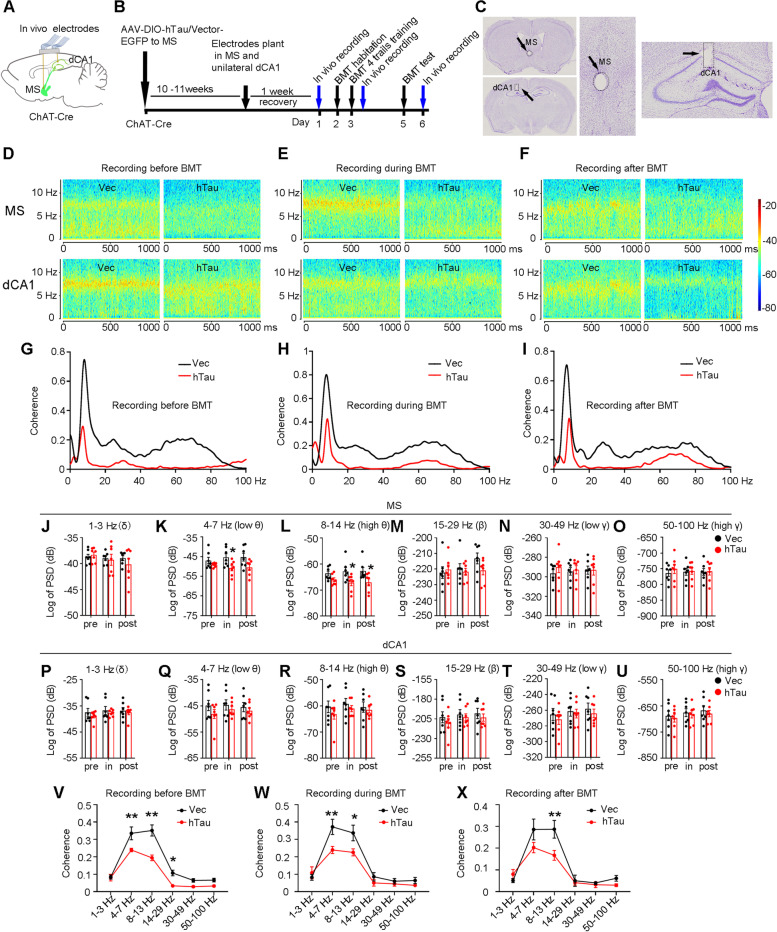
Cholinergic-specific overexpressing hTau disrupts theta rhythm in the MS and its synchronization with CA1. **A**, **B** Diagram and schematics shown local field potential signal recording in the MS and CA1. **C** Nissl staining confirmed electrode position in the MS and the unilateral CA1. **D**-**F** Representative heatmap of MS and dCA1 shown decreased power of theta rhythm measured before, during and after MBM test in MS-ChAT-hTau mice as compared with the control group. **G**-**I** Representative curves shown decreased coherence recorded in MS and CA1 of a MS-ChAT-hTau mouse and its control before, during and after MBM test. **J**-**O** Cholinergic-specific overexpressing hTau decreased theta power measured during (at 4–7 Hz and 8–14 Hz) and after (8–14 Hz) MBM test. **J** 1–3 Hz, unpaired t test, pre, t = 0.5813, df = 12, *P* > 0.05; in, t = 0.2973 df = 12, *P* > 0.05; post, t = 0.9549, df = 12, *P* > 0.05. **K** 4–7 Hz, unpaired t test, pre, t = 1.269, df = 12, *P* > 0.05; in, t = 2.378, df = 12, *P* < 0.05; post, t = 2.096, df = 12, *P* > 0.05. **L** 8 -14 Hz, unpaired t test, pre, t = 1.961 df = 12, *P* < 0.05; in, t = 2.262, df = 12, *P* < 0.05; post, t = 2.352, df = 12, *P* < 0.05. **M** 15–29 Hz, unpaired t test, pre, t = 0.4238, df = 12, *P* > 0.05; in, t = 0.4707, df = 12, *P* > 0.05; post, t = 1.684, df = 12, *P* > 0.05. **N** 30–49 Hz, unpaired t test, pre, t = 0.9446, df = 12, *P* > 0.05; in, t = 0.2927, df = 12, *P* > 0.05; post, t = 0.04679, df = 12, *P* > 0.05. **O** 50–100 Hz, unpaired t test, pre, t = 0.7450 df = 12, *P* > 0.05; in, t = 0.1598, df = 12, *P* > 0.05; post, t = 0.1340, df = 12, *P* > 0.05. **P**-**U** Cholinergic-specific overexpressing hTau did not significantly affect PSD in the CA1 at different frequency bands before, during and after BM test. **P** 1–3 Hz, unpaired t test, pre, t = 0.8001, df = 12, *P* > 0.05; in, t = 0.5970, df = 12, *P* > 0.05; post, t = 0.4382, df = 12, *P* > 0.05. **Q** 4–7 Hz, unpaired t test, pre, t = 1.245, df = 12, *P* > 0.05; in, t = 0.7448, df = 12, *P* > 0.05; post, t = 0.6663, df = 12, *P* > 0.05. **R** 8 -14 Hz, unpaired t test, pre, t = 0.9599 df = 12, *P* > 0.05; in, t = 0.7542, df = 12, *P* > 0.05; post, t = 0.4606, df = 12, *P* > 0.05. **S** 15–29 Hz, unpaired t test, pre, t = 0.7957, df = 12, *P* > 0.05; in, t = 0.4421, df = 12, *P* > 0.05; post, t = 0.6501, df = 12, *P* > 0.05. **T** 30–49 Hz, unpaired t test, pre, t = 0.6279, df = 12, *P* > 0.05; in, t = 0.3543, df = 12, *P* > 0.05; post, t = 0.6380, df = 12, *P* > 0.05. **U** 50–100 Hz, unpaired t test, pre, t = 0.3743, df = 12, *P* > 0.05; in, t = 0.3152, df = 12, *P* > 0.05; post, t = 0.5692 df = 12, *P* > 0.05. **V**-**X** Cholinergic-specific overexpressing hTau specifically disrupted theta coherence between MS and CA1 measured before, during and after BM test. **V** coherence, two–way ANOVA group × frequency, F [5,72] = 5.634, *P* < 0.01; **W** coherence, two–way ANOVA group × frequency, F [5,72] = 2.851, *P* < 0.05; **X** coherence, two–way ANOVA group × frequency, F [5,72] = 2.495, *P* < 0.05. *N* = 7 mice per group. *P > 0.05, ** *P* > 0.01. Data were presented as mean ± SEM

**Fig. 7:**
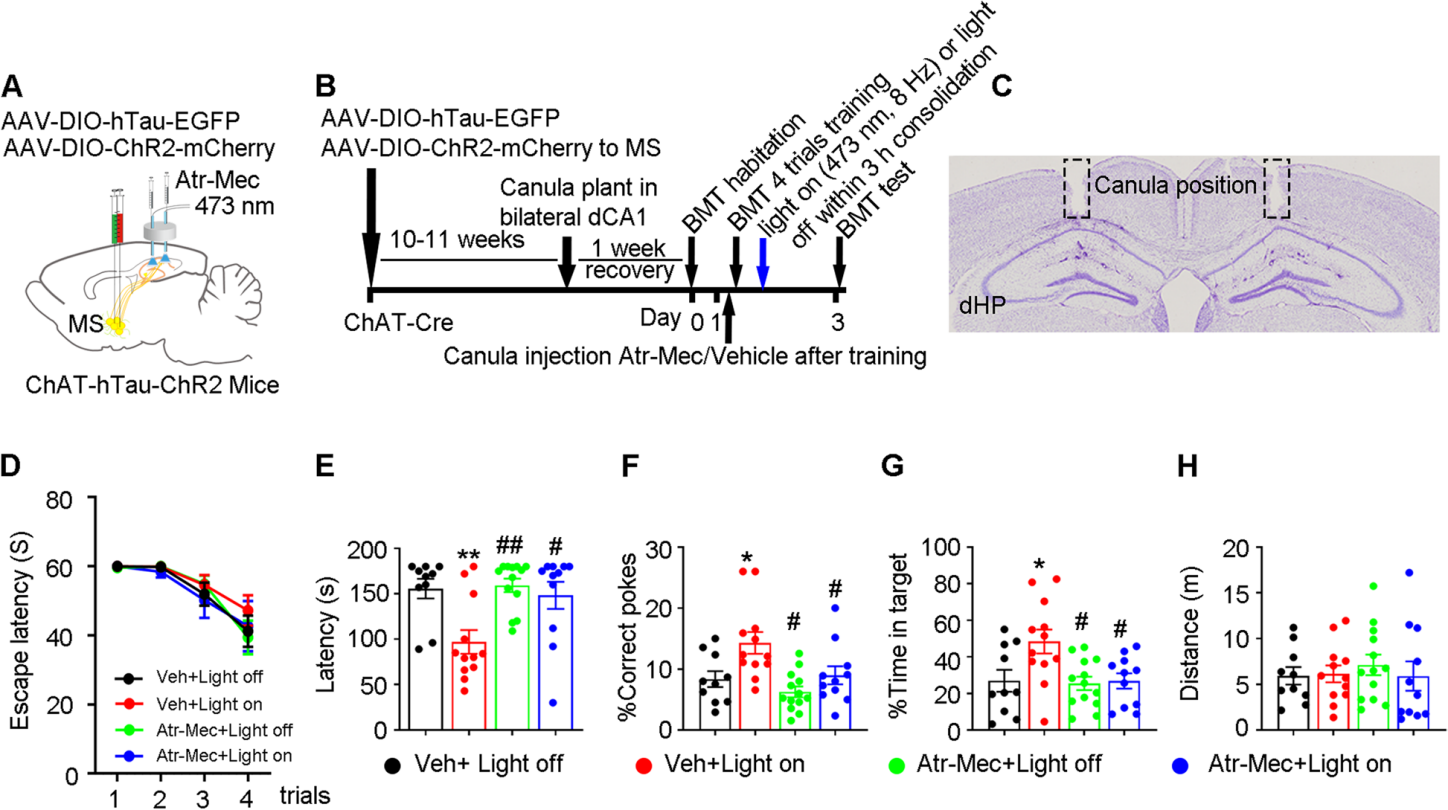
Optoactivating MS-CA1 cholinergic inputs at theta rhythm during critical consolidation time-window improves spatial memory in MS-ChAT-hTau mice. **A**, **B** Experiment diagram. In MS-ChAT-hTau mice, MS-CA1 cholinergic inputs was photoactivated with or without CA1 administration of acetylcholine receptor inhibitors. **C** Nissl staining confirmed the canula position in bilateral CA1. **D** During spatial training, no difference of escape latency was observed among groups (two–way ANOVA group × days, escape latency: F [9, 168] = 0.3797, *P* = 0.9436). **E** Optoactivation of MS-CA1 cholinergic inputs significantly decreased latency (**E** one–way ANOVA group, F [3, 42] = 6.404, *P* < 0.01) and increased correct pokes (**F** One–way ANOVA group, F [3, 42] = 6.422, *P* < 0.01) and time in the target (**G** One–way ANOVA group, F [3, 42] = 4.715 *P* < 0.01) in MS-ChAT-hTau mice. **E**–**G** The improvements were abolished when acetylcholine receptor inhibitors were injected into CA1 30 min before photostimulation. **H** No difference in moved distance was detected among the groups (**H** One–way ANOVA group, F [3, 42] = 0.2523, *P* > 0.05). *P < 0.05 vs Veh + Light off, **P < 0.01 vs Veh + Light off, ^#^*P* < 0.05, ^##^*P* < 0.01 vs: Veh + Light on. *N* = 10–13 mice per group. Data were presented as mean ± SEM

Finally, we searched for an efficient strategy to target the MS-CA1 cholinergic pathway to attenuate induced spatial memory loss by cholinergic tau accumulation. By in vivo electrical synchronous recording of the local field potential in MS and CA1 (Fig. 6A-C), we found that theta oscillation in the MS, but not in the dCA1, was dramatically suppressed in MS-ChAT-hTau mice during spatial training and memory consolidation phases in the MBM test (Fig. 6D-F, J-U). Simultaneously, theta coherence between the MS and dCA1 was significantly decreased in the MS-ChAT-hTau group compared with the controls (Fig. 6G-I, V-X). These data indicate that hTau accumulation in the MS disrupts theta rhythm in the MS and damages its synchronization with CA1, leading to spatial cognition-related communication dysfunction between the MS and hippocampal CA1. Then, we employed theta rhythm to photostimulate MS-CA1 cholinergic inputs within the memory consolidation phase during the MBM test (Fig. 7A-C). In line with the results from MS cholinergic activation, stimulating the MS-CA1 cholinergic circuit at 8 Hz could significantly decrease the latency (Fig. 7E), increase the correct pokes (Fig. 7F) and time in target (Fig. 7G) without changing the motor function in MS-ChAT-hTau mice (Fig. 7H), suggesting the beneficial effects of activating MS-CA1 cholinergic inputs on hTau-impaired memory capacity. Interestingly, the improvements were completely abolished by a prior dCA1 injection of blocker cocktails containing nicotinic and muscarinic acetylcholine receptor antagonists (Fig. 7E-H).

Together, these data indicate that the MS-CA1 cholinergic circuit is susceptible to hTau accumulation in the MS and that activating its cholinergic signaling at theta rhythm during the consolidation period (first 3 h after spatial training) could efficiently rescue hTau-induced memory dysfunction.

## Discussion

Cholinergic degeneration and abnormal tau accumulation are hallmark pathologies in the AD brain [31–33] that correlate with cognitive decline and disease severity [34]. However, the vulnerability of cholinergic neurons to AD-like tau accumulation and strategies to ameliorate tau-disrupted spatial memory in terms of neural circuits have not been fully elucidated. It is well known that the MS subset is rich in cholinergic neurons and hippocampal formation is in charge of the spatial cognitive function. Therefore, we conducted experiments to explore whether and how hTau accumulation in the cholinergic neurons of MS affect spatial learning and memory through the MS-hippocampus cholinergic circuit. We found that cholinergic hTau accumulation induced significant cholinergic neuron loss in the MS with a remarkably reduced cholinergic projection to the hippocampus. Functionally, hTau accumulation predominately inhibited the excitability of cholinergic neurons with asymmetric discharge characteristics in the MS-CA1 circuit and disrupted theta synchronization between the MS and CA1 during memory consolidation. Photostimulation of the MS-CA1 pathway within a critical 3 h memory consolidation time window efficiently improved spatial memory in a theta rhythm-dependent manner.

The basal forebrain is the primary niche for cholinergic neurons, which serves as the main source of cholinergic afferents in the hippocampus [35–39]. By in vitro patch clamp recordings, two cholinergic populations based on their distinct firing characteristics in the mature basal forebrain have been discovered. One subtype is early firing neurons (∼70%), which are easier to excite and more susceptible to depolarization blockade; the other subtype is late firing neurons (~ 30%) which are less excitable and maintain a tonic discharge at low frequencies [40]. As a subregion of the basal forebrain, whether cholinergic neurons in the MS are also heterogeneous in electrophysiology is still unknown. In the present study, we only detected the early firing subtype of cholinergic neurons in the MS according to the similarity of firing properties. Interestingly, two novel subtypes of cholinergic neurons were identified based on their symmetric (~ 22.9%) and asymmetric (~ 77.1%) firing characteristics. These results were in line with the findings from Li et al. [41]. By neuronal type-specific RNA-seq, they identified two types of cholinergic neurons in the MS, i.e., calbindin-D28K + (D28K +) and calbindin-D28K- (D28K-) [41]. *Cacna1h*, which encodes a low voltage-gated T-type calcium channel, determined the bursting activity of D28K- cholinergic neurons but had no effects on their frequency or threshold of action potential firing. However, *kcnh1,* which encodes the Eag1 potassium channel, controlled the frequency and threshold of action potential firing in D28K+ cholinergic neurons. Comparing our neural firing patterns with their data, we found that cholinergic neurons in the present study with symmetric firings and asymmetric firings were similar to their D28K- and D28K+ cholinergic neurons, respectively. Given the inhibition of cholinergic neurons with asymmetrical firing characteristics in the MS-CA1 circuit, we speculate that distinct molecules, such as calcium channels and potassium channels, may endow characteristics of symmetric and asymmetric cholinergic neurons in the MS and contribute to alterations in their neural excitability in response to tau accumulation. In addition, we found that ChAT+ neurons were different from CaMKII+ neurons in the MS. Given the crucial role of the MS-CA2 glutamatergic circuit in social memory [42], we speculate that distinct downstream targets may also contribute to the different behavior phenotypes between CaMKII+ and ChAT+ neurons in the MS. To the best of our knowledge, this is the first study to reveal the heterogeneity of cholinergic neurons in the MS from neuronal firing characteristics and the vulnerability to hTau toxicity in circuit specificity.

MS cholinergic neurons undergo intensive degeneration and loss during AD progression [43–45]. In AD animal models, a reduction in septal cholinergic neurons was detected by immunohistochemistry after β-amyloid (Aβ)1–40 or Aβ1-42 injection into the MS [46, 47]. Additionally, nonspecific ablation of neurons in the MS and vDB [48, 49] or selective elimination of their cholinergic neurons [50] could phenocopy AD-like spatial memory deficits, while administration of cholinesterase inhibitors was efficient in both animals and AD patients [12, 34, 51]. Tau pathologies have also been reported in cholinergic neurons in the AD brain [32, 52]. In THY-Tau22 mice, abundant phosphorylated tau was detected in the MS [53]. Currently, we found that hTau accumulation in the MS could directly cause cholinergic neuron loss and weaken cholinergic projections in a time-dependent manner, which identified the causal role of hTau in cholinergic lesions. Simultaneously, the excitability of cholinergic neurons was significantly inhibited by hTau accumulation in MS. Although DAPK [54], calcineurin [55], PKA [56], IST1 [57], and STAT1 [58] have been reported in hTau-induced neuron loss and synaptic plasticity dysfunction, the specific protein(s)/pathways contributing to cholinergic lesion upon hTau accumulation in the MS may deserve further identification.

The hippocampus receives cholinergic information flow from MS [58–60]. MS and hippocampus can orchestrate to modulate cognitive capacity via theta and gamma rhythms [61–63]. Several lines of evidence indicate that MS plays a critical role in hippocampal theta rhythm generation [64–66]. Furthermore, the occurrence of hippocampal theta rhythm depends on the proportion of septal neurons involved in the rhythmic process, while the frequency of theta field activity is determined by the frequency of rhythmical “theta” bursts in septal neurons [67–69]. Septal lesions, in addition to blocking theta, produce severe impairments in memory processes [69, 70]. In the present study, we dissected cholinergic projections from the MS to the hippocampal CA1 region by using an anterograde tracing system. More importantly, we also identified impaired theta synchronization between the MS and CA1 in MS-ChAT-hTau mice during memory consolidation. Our behavioral data demonstrated that impaired theta rhythm firing plays a key role in consolidation and spatial memory deficits produced by hTau accumulation in the MS. In addition to the previous basal forebrain-entorhinal pathway [38, 39, 71], the present study highlights MS-CA1 circuit and uncovers its essential role in AD memory impairments [72, 73] via consolidation mechanisms. Whether these parallel circuits are involved simultaneously or sequentially during the AD process and how they govern cognitive loss coordinately deserve further investigation.

In the present study, we revised the tradition Barnes maze as Wahlstrom et al. previously reported [18] to investigate memory consolidation mechanisms. The traditional Barnes maze consists of 4 days of training (4 trials per day) and a memory test. Notably, after the first day of training, the remaining 3 days of training sessions are inevitably interspersed with memorization components. Therefore, the traditional Barnes maze is not clean enough to investigate memory consolidation after a new learning. However, the modified Barnes maze, which consists of only 1-day training and a probe trial 2 days later, performed relatively well in two well-defined phases, i.e., a new learning phase and memory consolidation phase. After precisely stimulating MS-CA1 cholinergic inputs within the 3 h time window of consolidation, we found a significant improvement in spatial memory in MS-ChAT-hTau mice. In addition to the precise timing of targeting the MS-CA1 cholinergic pathway in the modified Barnes maze, the specific stimulating rhythm, 8 Hz, not 20 Hz, used in tradition Barnes maze, contributes to the beneficial effects on cholinergic tau accumulation-impaired spatial memory. Currently, traditional anti-AD medicines exhibit side effects and inefficiency, while optogenetic strategies exert neuron type, temporal and spatial precision. Therefore, after overcoming obstacles, such as invasion, autoimmunity, overheating from light stimulation, targeting the MS-CA1 pathway specifically within the 3 h time window after learning at 8 Hz prior to the hippocampal deficits produced by local tau pathology may benefit memory in patients with AD.

## Conclusion

Abnormal tau accumulation and cholinergic degeneration are hallmark pathologies in the brains of AD patients. However, the causal relationship between these two pathologies is not yet clear. Here, in the MS, we found a notable toxicity of tau accumulation in cholinergic neuron loss. Intriguingly, cholinergic neurons with an asymmetric discharge characteristic, especially in the MS-hippocampal CA1 circuit, were more vulnerable to tau accumulation and were responsible for tau-impaired spatial memory. Further, we identified that the first 3 h in memory consolidation and the theta rhythm photostimulation pattern are the two critical factors controlling the beneficial effects of targeting the MS-CA1 cholinergic pathway on tau-impaired spatial memory. Therefore, our study not only reveals the vulnerability of a novel MS-CA1 cholinergic circuit to AD-like tau accumulation, but also provides a rhythm- and time window-dependent strategy to target the MS-CA1 cholinergic circuit, thereby rescuing tau-induced spatial cognitive functions.

## Supplementary Information

**Additional file 1: sFigure 1.** Accumulation of hyperphosphorylated tau is remarkably increased in the medial septum (MS) of AD mouse models. (A-B) Representative images showing prominent accumulation of phosphorylated tau (pT205 and pT231) in the MS of 9-month 3xTg AD mice (A) and 5xFAD mice (B) measured by immunofluorescence staining. *N* = 3 mice per group. Scale bar, 50 μm.

**Additional file 2: sFigure 2.** Molecular characterization of cholinergic neurons in the MS. (A, D) Representative images show co-localization of ChAT with CaMKII or GABA by co-immunofluorescence staining. (B, C, E, F) Quantitative analyses showed that ~93% and ~28% of ChAT+ neurons were respectively co-stained with CaMKII and GABA, while ~13% CaMKII+ and ~7% GABA+ neurons were respectively ChAT. *N* = 6 mice per group. Scale bar, 50 μm.

**Additional file 3: sFigure 3.** Overexpressing hTau in CaMKII neuron does not induce spatial cognitive deficit or anxiety-related behaviors. (A, B) Overexpression of exogenous hTau in the CaMKII+ neurons of MS by infusion of AAV-CaMKII-Cre-mCherry and AAV-DIO-hTau/vector-EGFP, and ~95% of hTau were colocalized with CaMKII. *N* = 6 mice per group. Scale bar, 20 μm. (C-H) Three months after hTau overexpression, MS-CaMKII-hTau mice showed comparable spatial learning (C, D) and memory (E-H) with controls in BM test. During spatial learning trials, no differences of latency (C, two–way ANOVA group × days, escape latency: F [3,64] = 0.1376, *P *> 0.05) and number of errors (D, two–way ANOVA group × days, number of errors: F [3,64] = 0.11117, *P *> 0.05) were found between MS-CaMKII-hTau mice and the controls. In probe test, latency (E, unpaired *t* test, t = 0.7425 df = 16, *P *> 0.05), %correct poke (F, unpaired *t* test, t = 0.9169 df = 16, *P *> 0.05), %time in target (G, unpaired *t* test, t = 0.4458 df = 16, *P *> 0.05) and distance moved (H, unpaired *t* test, t = 0.01734 df = 16, *P *> 0.05) in MS-CaMKII-hTau group were identical to the controls. (I-N) Six months after hTau overexpression, MS-CaMKII-hTau mice displayed normal spatial learning (I, J) and memory (K-N) in BM test. I, two–way ANOVA group × days, escape latency: F [3,64] = 0.1346, *P *> 0.05; J, two–way ANOVA group × days, number of errors: F [3,64] = 0.3938, *P *> 0.05; K, unpaired *t* test, t = 0.5077 df = 16, *P *> 0.05, L, unpaired *t* test, t = 0.5866 df = 16, *P *> 0.05; M, unpaired *t* test, t = 0.1670 df = 16, *P *> 0.05 and N, unpaired *t* test, t = 0.1707 df = 16, *P *> 0.05. *N* = 9 mice per group (O-V) Overexpressing hTau in CaMKII+ neurons of MS for 3 or 6 m had no effects on anxiety-related behaviors in elevated plus maze test (O, P, S, T) and open field test (Q, R, U, V). O, unpaired *t* test, t = 0.5116 df = 18, *P* > 0.05 [3 m]; P, unpaired *t* test, t = 0.9208 df = 18, *P* > 0.05 [3 m]; S, unpaired *t* test, t = 0.3445 df = 18, *P* > 0.05 [6 m]; T, unpaired *t* test, t = 0.03656 df = 18, *P* > 0.05 [6 m]；Q, unpaired *t* test, t = 0.4622 df = 18, *P* > 0.05 [3 m]; R, unpaired *t* test, t = 0.02165 df = 18, *P* > 0.05 [3 m]; U, unpaired *t* test, t = 0.2111 df = 18, *P* > 0.05 [6 m]; V, unpaired *t* test, t = 0.2087 df = 18, *P* > 0.05 [6 m]. *N* = 10 mice per group. presented as mean ± SEM.

## Data Availability

Data supporting the results of this study are available from the corresponding author.

## Abbreviations

AD: Alzheimer’s disease
MS: Medial septum
HP: Hippocampus
CA1: Cornu Ammonis 1
dCA1: Dorsal Cornu Ammonis 1
PD: Parkinson disease
FTDP-17: Parkinsonsim-17
vDB: Vertical diagonal band of Broca
PCR: Polymerase chain reaction
DNA: Deoxy-ribonucleic acid
pAAV: Plasmid adeno-associated virus
DIO: Double-floxed inverse Orientation
eGFP: Enhanced green fluorescent protein
MAPT: Microtubule-associated protein Tau
ChR2: Channelrhodopsin-2
CaMKII: Ca2 + /calmodulin-dependent protein kinase II
eYFP: Enhanced yellow fluorescent protein
mCherry: Monomeric red fluorescent protein
AAV: Adeno-associated virus
ChAT: Choline acetyl transferase
GABA: Gamma-aminobutyric acid
CTB: Cholera toxin subunit B
CA3: Cornu Ammonis 3
AP: Anteroposterior
ML: Mediolateral
DV: Dorsoventral
DFT: Open-field test
NOR: Novel object recognition test
EPM: Elevated plus maze
BMT: Barnes maze test
DAPK: Death related protein kinase
PKA: Protein kinase A
IST1: IST1 factor associated with ESCRT-III
STAT1: Signal transducer and activator of transcription 1
