# Supplementary figures and images for: Targeting a vulnerable septum-hippocampus cholinergic circuit in a critical time window ameliorates tau-impaired memory consolidation
Source: Mol Neurodegener. 2023 Apr 14;18:23. doi: 10.1186/s13024-023-00614-7 (PMC10103508; doi:10.1186/s13024-023-00614-7)

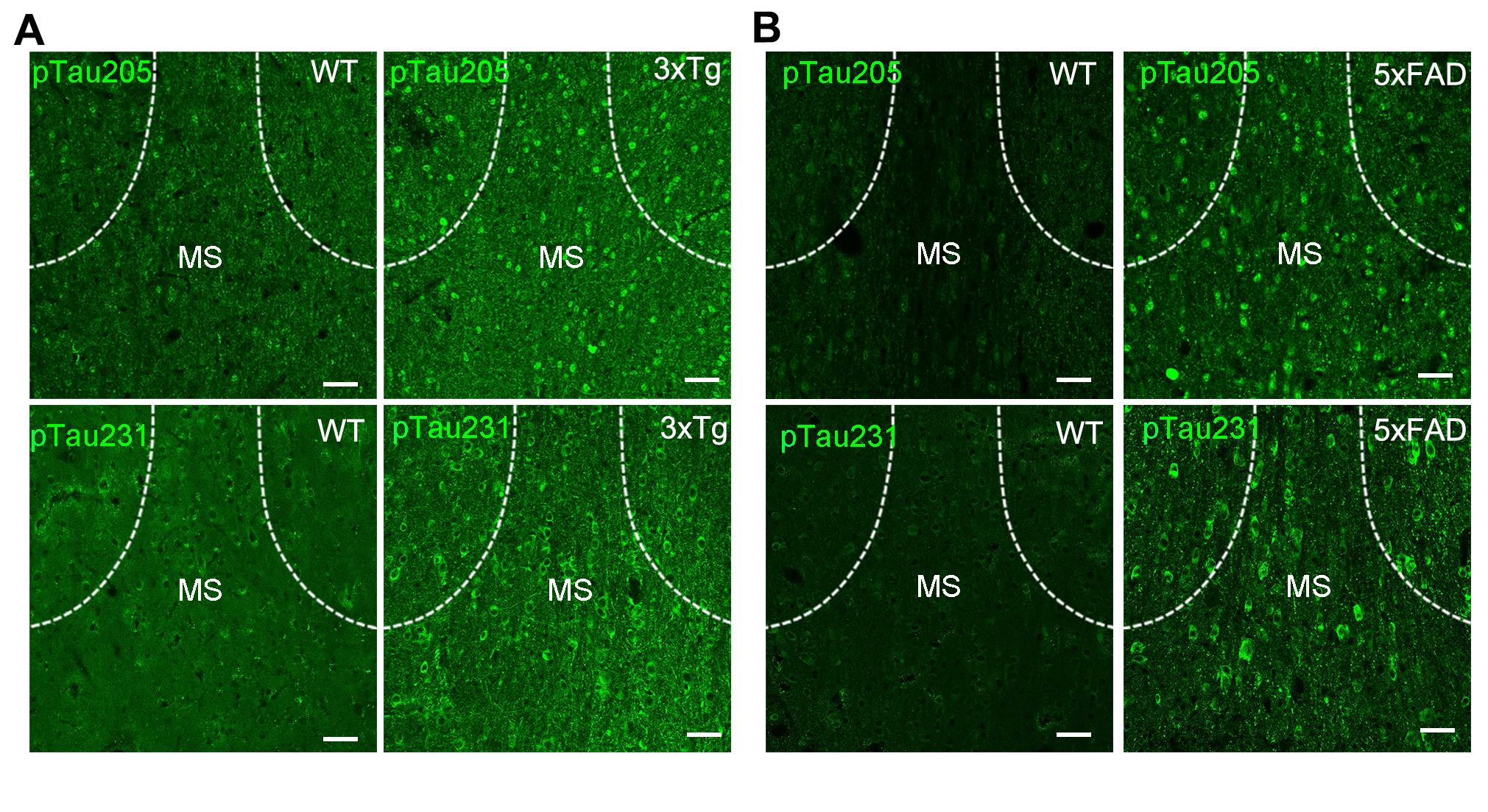

Supplement: Supplementary file 1 — Additional file 1: sFigure 1. Accumulation of hyperphosphorylated tau is remarkably increased in the medial septum (MS) of AD mouse models. (A-B) Representative images showing prominent accumulation of phosphorylated tau (pT205 and pT231) in the MS of 9-month 3xTg AD mice (A) and 5xFAD mice (B) measured by immunofluorescence staining. N = 3 mice per group. Scale bar, 50 μm. [file 13024_2023_614_MOESM1_ESM.tif]

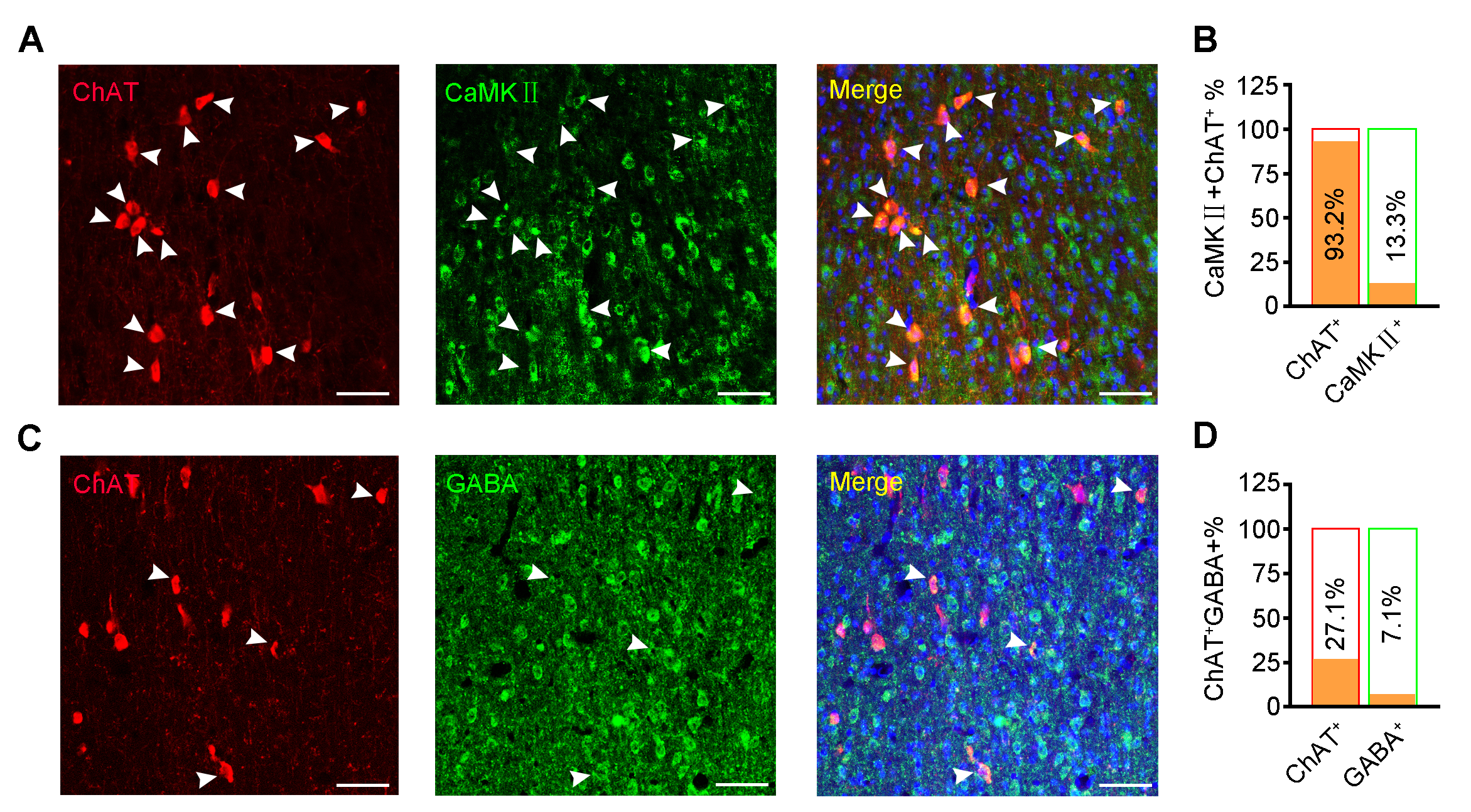

Supplement: Supplementary file 2 — Additional file 2: sFigure 2. Molecular characterization of cholinergic neurons in the MS. (A, D) Representative images show co-localization of ChAT with CaMKII or GABA by co-immunofluorescence staining. (B, C, E, F) Quantitative analyses showed that ~93% and ~28% of ChAT+ neurons were respectively co-stained with CaMKII and GABA, while ~13% CaMKII+ and ~7% GABA+ neurons were respectively ChAT. N = 6 mice per group. Scale bar, 50 μm. [file 13024_2023_614_MOESM2_ESM.tif]

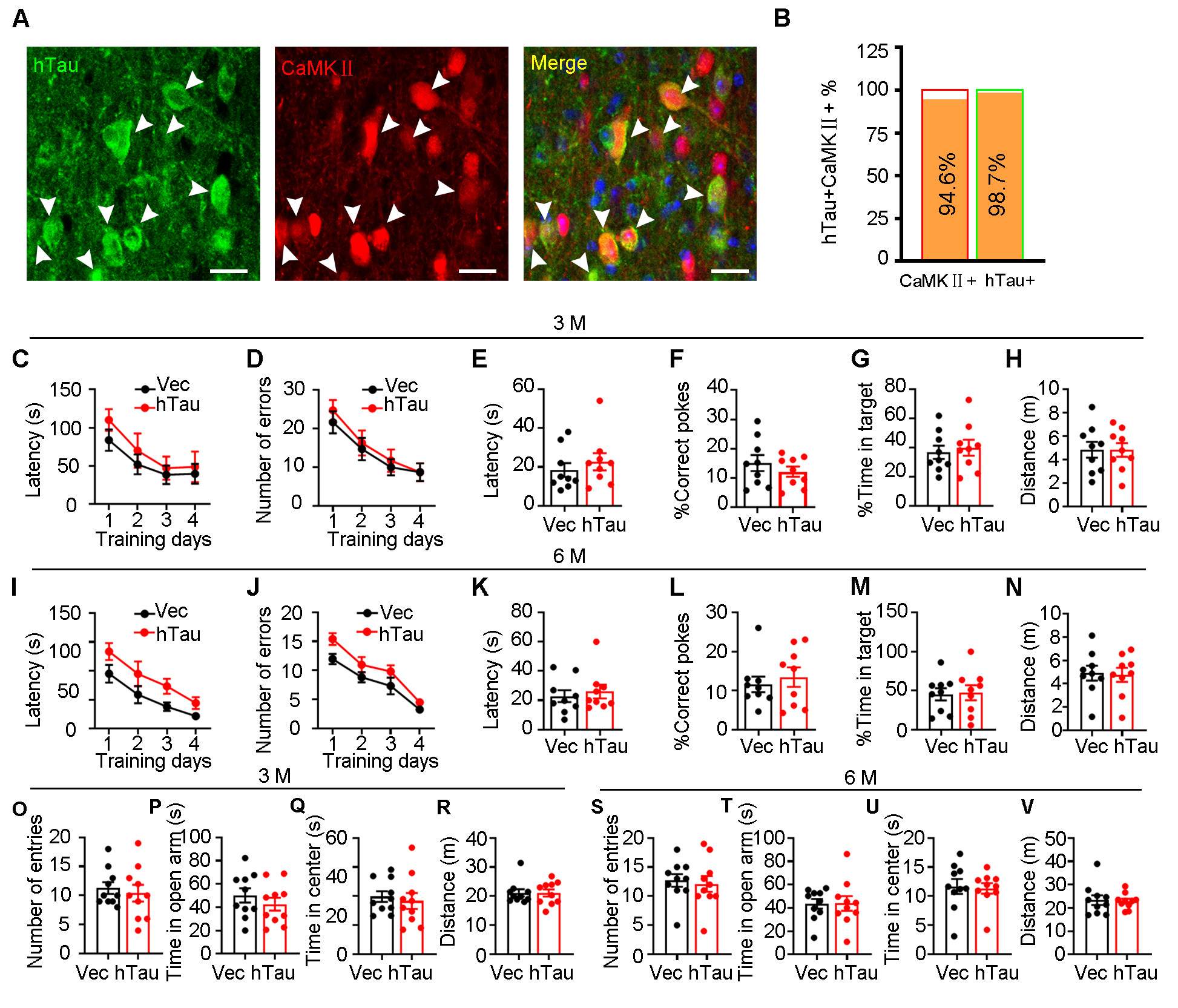

Supplement: Supplementary file 3 — Additional file 3: sFigure 3. Overexpressing hTau in CaMKII neuron does not induce spatial cognitive deficit or anxiety-related behaviors. (A, B) Overexpression of exogenous hTau in the CaMKII+ neurons of MS by infusion of AAV-CaMKII-Cre-mCherry and AAV-DIO-hTau/vector-EGFP, and ~95% of hTau were colocalized with CaMKII. N = 6 mice per group. Scale bar, 20 μm. (C-H) Three months after hTau overexpression, MS-CaMKII-hTau mice showed comparable spatial learning (C, D) and memory (E-H) with controls in BM test. During spatial learning trials, no differences of latency (C, two–way ANOVA group × days, escape latency: F [3,64] = 0.1376, P > 0.05) and number of errors (D, two–way ANOVA group × days, number of errors: F [3,64] = 0.11117, P > 0.05) were found between MS-CaMKII-hTau mice and the controls. In probe test, latency (E, unpaired t test, t = 0.7425 df = 16, P > 0.05), %correct poke (F, unpaired t test, t = 0.9169 df = 16, P > 0.05), %time in target (G, unpaired t test, t = 0.4458 df = 16, P > 0.05) and distance moved (H, unpaired t test, t = 0.01734 df = 16, P > 0.05) in MS-CaMKII-hTau group were identical to the controls. (I-N) Six months after hTau overexpression, MS-CaMKII-hTau mice displayed normal spatial learning (I, J) and memory (K-N) in BM test. I, two–way ANOVA group × days, escape latency: F [3,64] = 0.1346, P > 0.05; J, two–way ANOVA group × days, number of errors: F [3,64] = 0.3938, P > 0.05; K, unpaired t test, t = 0.5077 df = 16, P > 0.05, L, unpaired t test, t = 0.5866 df = 16, P > 0.05; M, unpaired t test, t = 0.1670 df = 16, P > 0.05 and N, unpaired t test, t = 0.1707 df = 16, P > 0.05. N = 9 mice per group (O-V) Overexpressing hTau in CaMKII+ neurons of MS for 3 or 6 m had no effects on anxiety-related behaviors in elevated plus maze test (O, P, S, T) and open field test (Q, R, U, V). O, unpaired t test, t = 0.5116 df = 18, P > 0.05 [3 m]; P, unpaired t test, t = 0.9208 df = 18, P > 0.05 [3 m]; S, unpaired t test, t = 0.3445 df = 18, P > 0.05 [6 [file 13024_2023_614_MOESM3_ESM.tif]
